# Supplementary figures and images for: Disruption of dopamine D2/D3 system function impairs the human ability to understand the mental states of other people
Source: PLoS Biol. 2024 Jun 13;22(6):e3002652. doi: 10.1371/journal.pbio.3002652 (PMC11175582; doi:10.1371/journal.pbio.3002652)

**
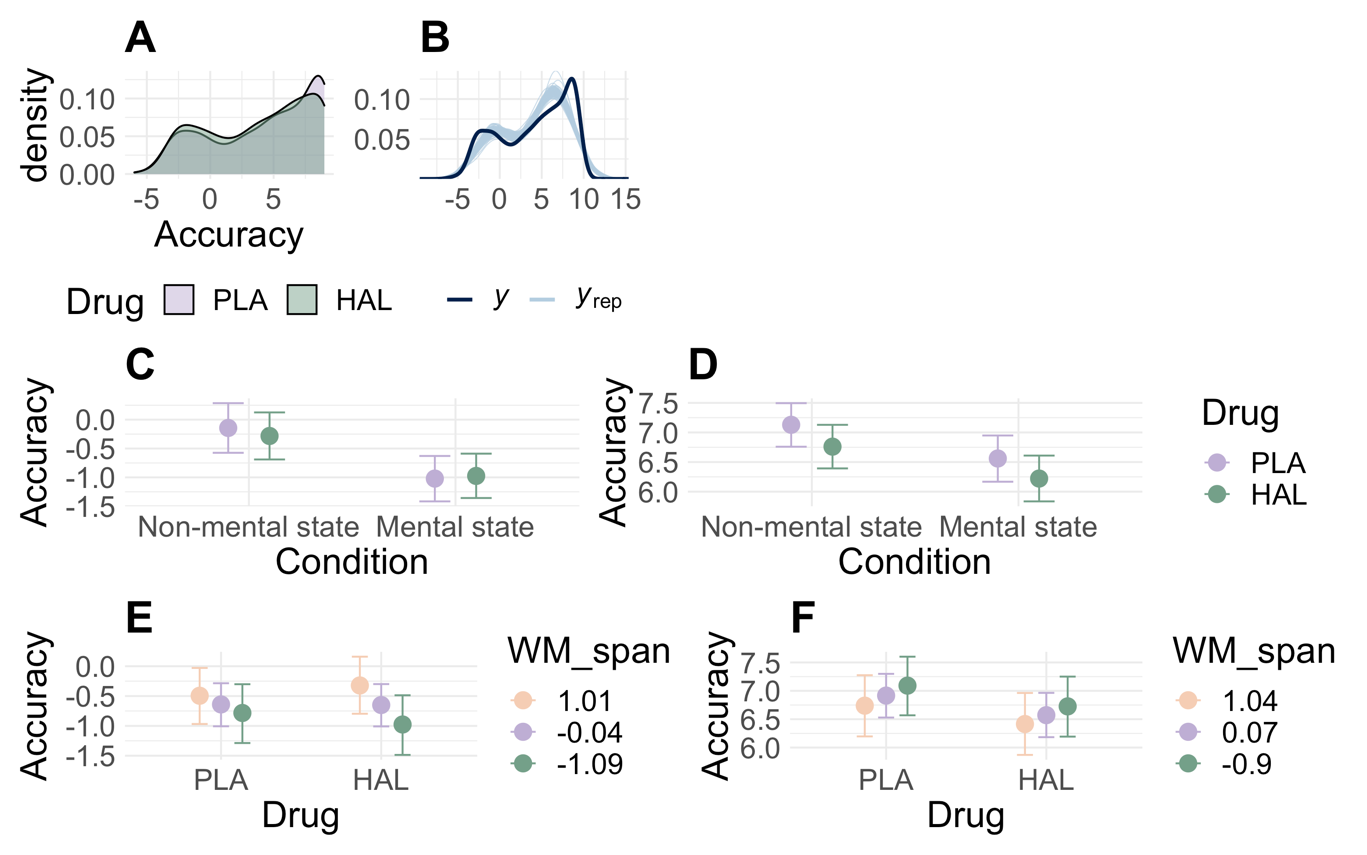
**

Supplement: S1 Fig — HAL, haloperidol trials; PLA, placebo trials. (A) Probability density plot of the response variable accuracy. (B) Posterior probability distribution of model 1.2. Y = response distribution, yrep = 100 draws from posterior samples. (C-F) Conditional effects plots (created using the “conditional_effects” function of the brms package [55]) for simple gaussian models fit to each distribution component individually, (C, D) depicting the interaction term of drug and mental state, (E, F) depicting the interaction term of drug and baseline WM. C = distribution component 1 comprising accuracy values < 3.01, D = distribution component 2 with accuracy values > 3.01. E = distribution component 1 comprising accuracy values < 3.01, E = distribution component 2 with accuracy values > 3.01. (DOCX) [file pbio.3002652.s001.docx]
