# Supplementary material for: Disruption of dopamine D2/D3 system function impairs the human ability to understand the mental states of other people
Source: PLoS Biol. 2024 Jun 13;22(6):e3002652. doi: 10.1371/journal.pbio.3002652 (PMC11175582; doi:10.1371/journal.pbio.3002652)
Supplement: S1 Tables — S1A Table. Model parameters for model 1.1. Model formula: accuracy ~ drug + (1 + drug || subject ID) + (1 | animation ID). S1B Table. Model parameters for model 1.2. Model formula: accuracy ~ drug * mental state + (1 + drug || subject ID) + (1 | animation ID). S1C Table. Model parameters for model 1.3. Model formula: accuracy ~ drug * drug day + (1 + drug || subject ID) + (1 | animation ID). S1D Table. Model parameters for model 1.4. Model formula: accuracy ~ drug * arousal + (1 + drug || subject ID) + (1 | animation ID). L = linear-, Q = quadratic-, C = cubic-, E4-E7 = fourth-seventh order polynomial trend. S1E Table. Leave-one-out (Loo) cross-comparison of models 1.1 and 1.4. Elpd_diff = Bayesian LOO estimate of the expected log pointwise predictive density (see [58]); se_diff = standard error of elpd_diff. Model weights were obtained using the brms function “model_weights.” (DOCX) [file pbio.3002652.s002.docx]

**S1A**

| Population-level effects | Estimate | Error | 95% CrI (lower) | 95% CrI (upper) |
| --- | --- | --- | --- | --- |
| *Intercept* | 4.27 | 0.27 | 3.73 | 4.81 |
| *HAL vs PLA* | -0.56 | 0.19 | -0.94 | -0.19 |
|  |  |  |  |  |
| Group-level effects | **Estimate (SD)** | **Error** | **95% CrI (lower)** | **95% CrI (upper)** |
| *Subject ID (Intercept)* | 1.13 | 0.16 | 0.86 | 1.47 |
| *Subject ID (drug)* | 0.68 | 0.22 | 0.20 | 1.12 |
| *Animation ID (Intercept)* | 2.20 | 0.15 | 1.92 | 2.51 |

**S1B**

| Population-level effects | Estimate | Error | 95% CrI (lower) | 95% CrI (upper) |
| --- | --- | --- | --- | --- |
| *Intercept* | 5.36 | 0.31 | 4.75 | 5.95 |
| *HAL vs PLA* | -0.66 | 0.23 | -1.12 | -0.20 |
| *Mental vs non-mental* | -2.50 | 0.37 | -3.22 | -1.78 |
| *HAL vs PLA, mental vs non-mental* | 0.20 | 0.28 | -0.34 | 0.74 |
|  |  |  |  |  |
| Group-level effects | **Estimate (SD)** | **Error** | **95% CrI (lower)** | **95% CrI (upper)** |
| *Subject ID (Intercept)* | 1.12 | 0.16 | 0.85 | 1.47 |
| *Subject ID (drug)* | 0.69 | 0.22 | 0.23 | 1.11 |
| *Animation ID (Intercept)* | 1.84 | 0.13 | 1.60 | 2.13 |

**S1C**

| Population-level effects | Estimate | Error | 95% CrI (lower) | 95% CrI (upper) |
| --- | --- | --- | --- | --- |
| *Intercept* | 4.21 | 0.36 | 3.50 | 4.92 |
| *HAL vs PLA* | -0.52 | 0.28 | -1.08 | 0.03 |
| *Drug day 1 vs 2* | 0.12 | 0.42 | -0.71 | 0.93 |
| *HAL vs PLA, Drug day 1 vs 2* | -0.06 | 0.38 | -0.81 | 0.68 |
|  |  |  |  |  |
| Group-level effects | **Estimate (SD)** | **Error** | **95% CrI (lower)** | **95% CrI (upper)** |
| *Subject ID (Intercept)* | 1.14 | 0.16 | 0.87 | 1.49 |
| *Subject ID (drug)* | 0.72 | 0.22 | 0.27 | 1.15 |
| *Animation ID (Intercept)* | 2.20 | 0.15 | 1.92 | 2.52 |

**S1D**

| Population-level effects | Estimate | Error | 95% CrI (lower) | 95% CrI (upper) |
| --- | --- | --- | --- | --- |
| *Intercept* | 4.34 | 0.32 | 3.72 | 4.96 |
| *HAL vs PLA* | -0.52 | 0.30 | -1.11 | 0.07 |
| *Arousal – L* | -0.21 | 0.71 | -1.61 | 1.18 |
| *Arousal – Q* | 0.02 | 0.70 | -1.37 | 1.38 |
| *Arousal – C* | -0.16 | 0.65 | -1.44 | 1.12 |
| *Arousal – E4* | 0.41 | 0.57 | -0.68 | 1.55 |
| *Arousal – E5* | 0.00 | 0.54 | -1.07 | 1.06 |
| *Arousal – E6* | 1.09 | 0.55 | -0.00 | 2.18 |
| *Arousal – E7* | 0.64 | 0.47 | -0.28 | 1.55 |
| *HAL vs PLA, arousal L* | -0.38 | 1.00 | -2.31 | 1.59 |
| *HAL vs PLA, arousal Q* | 0.48 | 0.89 | -1.28 | 2.22 |
| *HAL vs PLA, arousal C* | -0.25 | 0.87 | -1.94 | 1.47 |
| *HAL vs PLA, arousal E4* | -0.17 | 0.89 | -1.91 | 1.56 |
| *HAL vs PLA, arousal E5* | -0.33 | 0.83 | -1.96 | 1.31 |
| *HAL vs PLA, arousal E6* | -0.63 | 0.72 | -2.04 | 0.79 |
| *HAL vs PLA, arousal E7* | -1.50 | 0.58 | -2.61 | -0.36 |
|  |  |  |  |  |
| Group-level effects | **Estimate (SD)** | **Error** | **95% CrI (lower)** | **95% CrI (upper)** |
| *Subject ID (Intercept)* | 1.11 | 0.17 | 0.81 | 1.49 |
| *Subject ID (drug)* | 0.38 | 0.24 | 0.02 | 0.91 |
| *Animation ID (Intercept)* | 2.21 | 0.15 | 1.92 | 2.53 |

**S1E**

|  | elpd_diff | se_diff | Model weights |
| --- | --- | --- | --- |
| Model 1.4 | 0.0 | 0.0 | 0.52 |
| Model 1.1 | -0.2 | 2.9 | 0.47 |
