# Supplementary material for: Disruption of dopamine D2/D3 system function impairs the human ability to understand the mental states of other people
Source: PLoS Biol. 2024 Jun 13;22(6):e3002652. doi: 10.1371/journal.pbio.3002652 (PMC11175582; doi:10.1371/journal.pbio.3002652)
Supplement: S2 Tables — S2A Table. Model parameters for model 2.1. Model formula: accuracy ~ drug * mental state * jerk difference + (1 + drug || subject ID) + (1 | animation ID). S2B Table. Model parameters for model 2.2 (PLA only). Model formula: accuracy ~ jerk difference * mental state + (1 | subject ID) + (1 | animation ID). S2C Table. Model parameters for model 2.3 (HAL only). Model formula: accuracy ~ jerk difference * mental state + (1 | subject ID) + (1 | animation ID). (DOCX) [file pbio.3002652.s003.docx]

**S2A**

| Population-level effects | Estimate | Error | 95% CrI (lower) | 95% CrI (upper) |
| --- | --- | --- | --- | --- |
| *Intercept* | 5.39 | 0.30 | 4.81 | 5.99 |
| *HAL vs PLA* | -0.69 | 0.23 | -1.14 | -0.22 |
| *Mental vs non-mental* | -2.71 | 0.37 | -3.47 | -2.00 |
| *Jerk difference* | -0.11 | 0.13 | -0.36 | 0.14 |
| *HAL vs PLA, mental vs non-mental* | 0.44 | 0.31 | -0.17 | 1.06 |
| *HAL vs PLA, jerk difference* | 0.06 | 0.17 | -0.26 | 0.39 |
| *Mental vs non-mental, jerk difference* | -0.54 | 0.28 | -1.09 | -0.00 |
| *HAL vs PLA, mental vs non-mental, jerk difference* | 0.68 | 0.41 | -0.11 | 1.48 |
|  |  |  |  |  |
| Group-level effects | **Estimate (SD)** | **Error** | **95% CrI (lower)** | **95% CrI (upper)** |
| *Subject ID (Intercept)* | 1.09 | 0.15 | 0.82 | 1.43 |
| *Subject ID (drug)* | 0.66 | 0.22 | 0.20 | 1.08 |
| *Animation ID (Intercept)* | 1.81 | 0.14 | 1.56 | 2.09 |

**S2B**

| Population-level effects | Estimate | Error | 95% CrI (lower) | 95% CrI (upper) |
| --- | --- | --- | --- | --- |
| *Intercept* | 5.39 | 0.30 | 4.80 | 5.98 |
| *Jerk difference* | -0.13 | 0.14 | -0.41 | 0.14 |
| *Mental vs non-mental* | -2.76 | 0.37 | -3.47 | -2.03 |
| *Jerk difference, mental vs non-mental* | -0.70 | 0.31 | -1.32 | -0.09 |
|  |  |  |  |  |
| Group-level effects | **Estimate (SD)** | **Error** | **95% CrI (lower)** | **95% CrI (upper)** |
| *Subject ID (Intercept)* | 1.07 | 0.18 | 0.76 | 1.48 |
| *Animation ID (Intercept)* | 1.72 | 0.16 | 1.42 | 2.05 |

**S2C**

| Population-level effects | Estimate | Error | 95% CrI (lower) | 95% CrI (upper) |
| --- | --- | --- | --- | --- |
| *Intercept* | 4.74 | 0.32 | 4.10 | 5.36 |
| *Jerk difference* | -0.10 | 0.14 | -0.37 | 0.18 |
| *Mental vs non-mental* | -2.26 | 0.38 | -2.99 | -1.52 |
| *Jerk difference, mental vs non-mental* | 0.02 | 0.36 | -0.69 | 0.73 |
|  |  |  |  |  |
| Group-level effects | **Estimate (SD)** | **Error** | **95% CrI (lower)** | **95% CrI (upper)** |
| *Subject ID (Intercept)* | 1.36 | 0.19 | 1.02 | 1.78 |
| *Animation ID (Intercept)* | 1.74 | 0.16 | 1.46 | 2.07 |
