# Supplementary material for: Disruption of dopamine D2/D3 system function impairs the human ability to understand the mental states of other people
Source: PLoS Biol. 2024 Jun 13;22(6):e3002652. doi: 10.1371/journal.pbio.3002652 (PMC11175582; doi:10.1371/journal.pbio.3002652)
Supplement: S3 Tables — S3A Table. Model parameters for model 3.1. Model formula: accuracy ~ drug * mental state * PLA jerk difference + (1 + drug || subject ID) + (1 | animation ID). S3B Table. Model parameters for model 3.2. Model formula: accuracy ~ mental state * PLA jerk difference * HAL jerk difference + (1 + subject ID) + (1 | animation ID). (DOCX) [file pbio.3002652.s004.docx]

**S3A**

| Population-level effects | Estimate | Error | 95% CrI (lower) | 95% CrI (upper) |
| --- | --- | --- | --- | --- |
| *Intercept* | 5.39 | 0.30 | 4.79 | 5.98 |
| *HAL vs PLA* | -0.74 | 0.25 | -1.23 | -0.26 |
| *Mental vs non-mental* | -2.75 | 0.37 | -3.47 | -2.04 |
| *PLA jerk difference* | -0.16 | 0.13 | -0.42 | 0.10 |
| *HAL vs PLA, mental vs non-mental* | 0.35 | 0.31 | -0.26 | 0.96 |
| *HAL vs PLA, PLA jerk difference* | 0.16 | 0.17 | -0.18 | 0.50 |
| *Mental vs non-mental, PLA jerk difference* | -0.55 | 0.28 | -1.10 | 0.00 |
| *HAL vs PLA, mental vs non-mental, PLA jerk difference* | 0.19 | 0.38 | -0.56 | 0.94 |
|  |  |  |  |  |
| Group-level effects | **Estimate (SD)** | **Error** | **95% CrI (lower)** | **95% CrI (upper)** |
| *Subject ID (Intercept)* | 1.15 | 0.18 | 0.85 | 1.53 |
| *Subject ID (drug)* | 0.74 | 0.23 | 0.25 | 1.21 |
| *Animation ID (Intercept)* | 1.78 | 0.14 | 1.53 | 2.06 |

**S3B**

| Population-level effects | Estimate | Error | 95% CrI (lower) | 95% CrI (upper) |
| --- | --- | --- | --- | --- |
| *Intercept* | 4.67 | 0.35 | 3.98 | 5.35 |
| *Mental vs non-mental* | -2.33 | 0.39 | -3.10 | -1.55 |
| *PLA jerk difference* | -0.06 | 0.19 | -0.44 | 0.31 |
| *HAL Jerk difference* | 0.03 | 0.19 | -0.35 | 0.40 |
| *Mental vs non-mental, PLA jerk difference* | -0.70 | 0.35 | -1.38 | -0.03 |
| *Mental vs non-mental, HAL jerk difference* | -0.30 | 0.42 | -1.14 | 0.50 |
| *PLA jerk difference x HAL jerk difference* | -0.02 | 0.09 | -0.21 | 0.16 |
| *Mental vs non-mental, PLA jerk difference x HAL jerk difference* | -1.01 | 0.45 | -1.89 | -0.14 |
|  |  |  |  |  |
| Group-level effects | **Estimate (SD)** | **Error** | **95% CrI (lower)** | **95% CrI (upper)** |
| *Subject ID (Intercept)* | 1.45 | 0.16 | 1.44 | 2.08 |
| *Animation ID (Intercept)* | 1.75 | 0.23 | 1.07 | 1.95 |
