# Supplementary material for: Disruption of dopamine D2/D3 system function impairs the human ability to understand the mental states of other people
Source: PLoS Biol. 2024 Jun 13;22(6):e3002652. doi: 10.1371/journal.pbio.3002652 (PMC11175582; doi:10.1371/journal.pbio.3002652)
Supplement: S4 Tables — S4A Table. Model parameters for model 4.1. Model formula: accuracy change | trunc(lb = −1, ub = 1) ~ emotion change * mental state + WM change * mental state. ER change = emotion recognition change; WM change = working memory change. S4B Table. Leave-one-out (loo) cross-comparison of models 4.1 and 4.1.rand. Elpd_diff = Bayesian LOO estimate of the expected log pointwise predictive density (see [58]); se_diff = standard error of elpd_diff. Model weights were obtained using the brms function “model_weights.” S4C Table. Leave-one-out (loo) cross-comparison of models 4.1 and 4.1.cum. Elpd_diff = Bayesian LOO estimate of the expected log pointwise predictive density (see [58]); se_diff = standard error of elpd_diff. S4D Table. Model parameters for model 4.2. Model formula: accuracy change | trunc(lb = −1, ub = 1) ~ ER change * mental state. ER change = emotion recognition change. (DOCX) [file pbio.3002652.s005.docx]

**S4A**

| Population-level effects | Estimate | Error | 95% CrI (lower) | 95% CrI (upper) |
| --- | --- | --- | --- | --- |
| *Intercept* | -0.05 | 0.02 | -0.10 | -0.00 |
| *ER change* | -0.02 | 0.02 | -0.07 | 0.03 |
| *Mental vs non-mental* | -0.02 | 0.03 | -0.08 | 0.05 |
| *WM change* | -0.00 | 0.00 | -0.01 | 0.01 |
| *ER change, mental vs non-mental* | 0.06 | 0.03 | -0.00 | 0.13 |
| *WM change, mental vs non-mental* | -0.01 | 0.00 | -0.02 | 0.00 |

**S4B**

|  | elpd_diff | se_diff | Model weights |
| --- | --- | --- | --- |
| Model 4.1 | 0.0 | 0.0 | 1 |
| Model 4.1.rand | -0.7 | 0.8 | 0 |

**S4C**

|  | elpd_diff | se_diff |
| --- | --- | --- |
| Model 4.1 | 0.0 | 0.0 |
| Model 4.1.cum | -33.9 | 6.1 |

**S4D**

| Population-level effects | Estimate | Error | 95% CrI (lower) | 95% CrI (upper) |
| --- | --- | --- | --- | --- |
| *Intercept* | -0.05 | 0.02 | -0.09 | -0.00 |
| *ER change* | -0.02 | 0.02 | -0.07 | 0.03 |
| *Mental vs non-mental* | -0.01 | 0.03 | -0.08 | 0.06 |
| *ER change, mental vs non-mental* | 0.07 | 0.03 | -0.00 | 0.13 |
