# Supplementary material for: Disruption of dopamine D2/D3 system function impairs the human ability to understand the mental states of other people
Source: PLoS Biol. 2024 Jun 13;22(6):e3002652. doi: 10.1371/journal.pbio.3002652 (PMC11175582; doi:10.1371/journal.pbio.3002652)
Supplement: S5 Tables — S5A Table. Model parameters for model 5. Model formula: accuracy ~ drug * mental state + (1 | subject ID). Response modelled as a mixture of 2 gaussian distributions. S5B Table. Model parameters for model 6.1. Model formula: accuracy ~ drug * WM + (1 | subject ID). WM = working memory; response modelled as a mixture of 2 gaussian distributions. S5C Table. Model parameters for model 6.2 (post hoc model—low WM). Model formula: accuracy ~ drug + (1 | subject ID). Response modelled as a mixture of 2 gaussian distributions. S5D Table. Model parameters for model 6.3 (post hoc model—high WM). Model formula: accuracy ~ drug + (1 | subject ID). Response modelled as a mixture of 2 gaussian distributions. (DOCX) [file pbio.3002652.s006.docx]

**S5A**

| Population-level effects | Estimate | Error | 95% CrI (lower) | 95% CrI (upper) |
| --- | --- | --- | --- | --- |
| *Intercept_1_* | -0.42 | 0.22 | -0.86 | 0.01 |
| *Intercept_2_* | 6.87 | 0.17 | 6.54 | 7.19 |
| *HAL vs PLA_1_* | -0.25 | 0.27 | -0.76 | 0.28 |
| *Mental vs non-mental_1_* | -1.17 | 0.24 | -1.63 | -0.70 |
| *HAL vs PLA, mental vs non-mental_1_* | 0.29 | 0.32 | -0.35 | 0.92 |
| *HAL vs PLA_2_* | -0.50 | 0.13 | -0.76 | -0.25 |
| *Mental vs non-mental_2_* | -0.81 | 0.14 | -1.09 | -0.53 |
| *HAL vs PLA, mental vs non-mental_2_* | 0.00 | 0.20 | -0.38 | 0.39 |
|  |  |  |  |  |
| Group-level effects | **Estimate (SD)** | **Error** | **95% CrI (lower)** | **95% CrI (upper)** |
| *Subject ID (Intercept_1_)* | 0.82 | 0.15 | 0.56 | 1.15 |
| *Subject ID (Intercept_2_)* | 1.35 | 0.17 | 1.07 | 1.72 |

**S5B**

| Population-level effects | Estimate | Error | 95% CrI (lower) | 95% CrI (upper) |
| --- | --- | --- | --- | --- |
| *Intercept_1_* | -1.08 | 0.16 | -1.39 | -0.78 |
| *Intercept_2_* | 6.49 | 0.17 | 6.16 | 6.82 |
| *HAL vs PLA_1_* | -0.02 | 0.17 | -0.36 | 0.32 |
| *Low_WM_1_* | -0.04 | 0.18 | -0.39 | 0.30 |
| *High_WM_1_* | 0.04 | 0.18 | -0.30 | 0.39 |
| *HAL vs PLA, low_WM_1_* | -0.34 | 0.18 | -0.68 | 0.01 |
| *HAL vs PLA, high_WM_1_* | 0.34 | 0.18 | 0.01 | -0.68 |
| *HAL vs PLA_2_* | -0.52 | 0.11 | -0.73 | -0.31 |
| *Low_WM_2_* | 0.30 | 0.26 | -0.21 | 0.80 |
| *High_WM_2_* | -0.30 | 0.26 | -0.21 | 0.80 |
| *HAL vs PLA, low_WM_2_* | -0.30 | 0.11 | -0.51 | -0.10 |
| *HAL vs PLA, high_WM_2_* | 0.30 | 0.11 | 0.10 | 0.51 |
|  |  |  |  |  |
| Group-level effects | **Estimate (SD)** | **Error** | **95% CrI (lower)** | **95% CrI (upper)** |
| *Subject ID (Intercept_1_)* | 0.76 | 0.15 | 0.50 | 1.07 |
| *Subject ID (Intercept_2_)* | 1.51 | 0.20 | 1.18 | 1.95 |

**S5C**

| Population-level effects | Estimate | Error | 95% CrI (lower) | 95% CrI (upper) |
| --- | --- | --- | --- | --- |
| *Intercept_1_* | -1.24 | 0.18 | -1.60 | -0.88 |
| *Intercept_2_* | 6.53 | 0.20 | 6.14 | 6.91 |
| *HAL vs PLA_1_* | -0.37 | 0.22 | -0.81 | 0.06 |
| *HAL vs PLA_2_* | -0.83 | 0.17 | -1.16 | -0.50 |
|  |  |  |  |  |
| Group-level effects | **Estimate (SD)** | **Error** | **95% CrI (lower)** | **95% CrI (upper)** |
| *Subject ID (Intercept_1_)* | 0.80 | 0.20 | 0.48 | 1.24 |
| *Subject ID (Intercept_2_)* | 1.69 | 0.31 | 1.19 | 2.41 |

**S5D**

| Population-level effects | Estimate | Error | 95% CrI (lower) | 95% CrI (upper) |
| --- | --- | --- | --- | --- |
| *Intercept_1_* | -1.20 | 0.23 | -1.66 | -0.76 |
| *Intercept_2_* | 6.24 | 0.19 | 5.87 | 6.60 |
| *HAL vs PLA_1_* | 0.21 | 0.29 | -0.36 | 0.77 |
| *HAL vs PLA_2_* | -0.21 | 0.13 | -0.46 | 0.04 |
|  |  |  |  |  |
| Group-level effects | **Estimate (SD)** | **Error** | **95% CrI (lower)** | **95% CrI (upper)** |
| *Subject ID (Intercept_1_)* | 1.04 | 0.33 | 0.48 | 1.79 |
| *Subject ID (Intercept_2_)* | 1.54 | 0.30 | 1.09 | 2.24 |
