# Supplementary material for: Disruption of dopamine D2/D3 system function impairs the human ability to understand the mental states of other people
Source: PLoS Biol. 2024 Jun 13;22(6):e3002652. doi: 10.1371/journal.pbio.3002652 (PMC11175582; doi:10.1371/journal.pbio.3002652)
Supplement: S6 Tables — S6A Table. Model parameters for model 7.1. Model formula: speed ~ drug * WM + (1 | subject ID). S6B Table. Model parameters for model 7.2 (post hoc model—low WM). Model formula: speed ~ drug * WM + (1 | subject ID). (DOCX) [file pbio.3002652.s007.docx]

**S6A**

| Population-level effects | Estimate | Error | 95% CrI (lower) | 95% CrI (upper) |
| --- | --- | --- | --- | --- |
| *Intercept* | 1.09 | 0.03 | 1.04 | 1.14 |
| *HAL vs PLA* | -0.04 | 0.02 | -0.08 | 0.01 |
| *Low WM* | -0.04 | 0.03 | -0.09 | 0.01 |
| *High WM* | 0.04 | 0.03 | -0.01 | 0.09 |
| *HAL vs PLA, low WM* | -0.04 | 0.02 | -0.09 | 0.00 |
| *HAL vs PLA, high WM* | 0.04 | 0.02 | 0.00 | 0.09 |
|  |  |  |  |  |
| Group-level effects | **Estimate (SD)** | **Error** | **95% CrI (lower)** | **95% CrI (upper)** |
| *Subject ID (Intercept)* | 0.14 | 0.02 | 0.11 | 0.18 |

**S6B**

| Population-level effects | Estimate | Error | 95% CrI (lower) | 95% CrI (upper) |
| --- | --- | --- | --- | --- |
| *Intercept* | 1.05 | 0.04 | 0.97 | 1.13 |
| *HAL vs PLA* | -0.08 | 0.04 | -0.16 | -0.01 |
|  |  |  |  |  |
| Group-level effects | **Estimate (SD)** | **Error** | **95% CrI (lower)** | **95% CrI (upper)** |
| *Subject ID (Intercept)* | 0.12 | 0.04 | 0.04 | 0.20 |
