# Supplementary material for: Disruption of dopamine D2/D3 system function impairs the human ability to understand the mental states of other people
Source: PLoS Biol. 2024 Jun 13;22(6):e3002652. doi: 10.1371/journal.pbio.3002652 (PMC11175582; doi:10.1371/journal.pbio.3002652)
Supplement: S1 Text — (DOCX) [file pbio.3002652.s008.docx]

**Inclusion criteria**

- Participant is willing and able to give informed consent for participation in the study
- Sufficient English to be able to consent and understand study instructions
- Aged 18-45
- BMI in range of 18.5 – 29.5

**Exclusion criteria**

- Participated in another drug study within last 3 weeks
- Primary sensory impairment (e.g., uncorrected visual or hearing impairment)
- Personal or first-degree relative history of: cardiovascular disease (specifically hypotension, arrhythmias or valvular disease, stroke)
- Personal history of: any neurological abnormalities or past traumas, kidney disease or liver disease, stomach ulcers, skin conditions, endocrine conditions,
- Inherited blood conditions
- Psychiatric or psychological disorder (e.g., depression, anxiety)
- Known learning disability
- Elongated Q-T interval identified during the health screening using single-lead ECG (heart rate corrected): > .420 ms
- Low heart rate
- Low or high blood pressure (outside of lower bound 90/60 – upper bound 140/90)
- Blood oxygenation below 95%
- Any regular medication (excluding oral contraceptive pill)
- Recent recreational drug use (within last 7 days) or alcohol and/or drug dependency
- Known allergy to any medication or lactose sensitivity
- Current pregnancy or breastfeeding

**Eligibility as confirmed by medic during health screening:**

During the health screening, participant and medic discussed the participant’s medical history by going through a health check questionnaire which participants filled out beforehand. Subsequently, a number of physiological measures were taken to confirm eligibility (e.g., cardiovascular and respiratory assessments, BMI, blood oxygenation %, resting blood pressure, resting heart rate ECG QT-interval).
