# Supplementary material for: Disruption of dopamine D2/D3 system function impairs the human ability to understand the mental states of other people
Source: PLoS Biol. 2024 Jun 13;22(6):e3002652. doi: 10.1371/journal.pbio.3002652 (PMC11175582; doi:10.1371/journal.pbio.3002652)
Supplement: S1 Results — (DOCX) [file pbio.3002652.s009.docx]

**Modelling the bimodality of the response**

Due to bimodality of the response variable (see S1A Fig), model 1.2 was re-run (Model 5, without fitting a random slope for animation ID due to convergence issues), this time modelling the response variable as a mixture of two gaussian distributions. S1B Fig shows that this captured the response structure reasonably well (note that a mixture of two skew-normal distributions would have provided an even better posterior fit but was not chosen due to convergence problems and to avoid overfitting). This approach resulted in a set of parameters for each of the two modelled distribution components. The model revealed a main effect of drug for the second distribution component (${E\mu}_{2,HALvsPLA, non-mental}$ = -0.50, CrI = [-0.76, -0.25]; ${E\mu}_{2,HALvsPLA, mentalVSnon-mental}$ = 0.00, CrI = [-0.38, 0.39]), comprising all accuracy values above the distribution intersection point of 3.01 (determined using the uniroot.all function of the rootSolve package^1^). As in model 1.2, there was no interaction of drug and mental state for either of the distribution components (see S1C-D Fig; S5A Table). Thus, after taking into account the bimodality of the response variable, the model shows the same negative effect of drug on accuracy for mental- and non-mental state animations, whereby this model highlights that haloperidol affected accuracy particularly for animations that were initially decoded with higher accuracy. Importantly, we show below that dopamine challenge also affected performance for the more difficult animations (first distribution component), but here likely via different mechanisms. Finally, for both distribution components, the model revealed the same main effect of mental state as model 1.2 (${E\mu}_{1,mentalVSnon-mental}$ = -1.17, CrI = [-1.63, -0.70]; ${E\mu}_{2,mentalVSnon-mental}$ = -0.81, CrI = [-1.09, -0.53]).

Based on previous evidence indicating that working memory span reliably predicts individual dopamine synthesis capacity^2,3^ and drug effects of working memory span on performance in other socio-cognitive^4^ and cognitive^5-9^ domains, we used individual baseline working memory span as a proxy for baseline dopamine function, to account for interindividual differences in drug responsivity. For this, working memory task accuracy was calculated as the percentage of correct responses of all placebo trials. Participants were then divided into groups of low and high baseline working memory span by performing a median split on working memory span scores from the placebo day (indexing low and high dopamine synthesis capacity, respectively; 18 subjects in the low, 20 in the high working memory group).

A second mixture model (Model 6.1, see S5B Table was fit to drug and *baseline working memory* (low, high; effects coded), as well as their interaction, predicting accuracy. This second model revealed an interaction between baseline working memory and drug for both distribution components, indicating a stronger decrease in the low, relative to the high WM group (${E\mu}_{1,HALvsPLA, lowWM}$ = -0.34, CrI = [-0.68, 0.01], ${E\mu}_{1,HALvsPLA, highWM}$ = 0.34, CrI = [-0.01, 0.68]; ${E\mu}_{2,HALvsPLA, lowWM}$ = -0.30, CrI = [-0.51, -0.10], ${E\mu}_{2,HALvsPLA, highWM}$ = 0.30, CrI = [0.10, 0.51]). Two separate post-hoc models for low and high WM groups confirm a negative effect of drug on accuracy for both distribution components in low WM individuals (Model 6.2 / S5C: ${E\mu}_{1,HALvsPLA}$ = -0.37, CrI = [-0.81, 0.06], ${E\mu}_{2,HALvsPLA}$ = -0.83, CrI = [-1.16, -0.50]), whereas the high WM group exhibited a negative drug effect for the second, but not the first distribution component (Model 6.3 / S5D: ${E\mu}_{1,HALvsPLA}$ = 0.21, CrI = [-0.36, 0.77], ${E\mu}_{2,HALvsPLA}$ = -0.21, CrI = [-0.46, 0.04]; see S1E-F Fig). Thus, for those animations classified with accuracy values below 3.01, the effect of haloperidol on accuracy depended on individuals’ baseline working memory capacity, whereby only individuals with low WM, and thus estimated low striatal dopamine synthesis capacity, showed decreased accuracy as a response to the drug. In contrast, both WM groups exhibited a decrease in accuracy after haloperidol for the animations with accuracy values above 3.01, albeit with a somewhat stronger negative effect in the low WM group.

1 rootSolve: Nonlinear root finding, equilibrium and

steady-state analysis of ordinary differential equations (2009).

2 Cools, R., Gibbs, S. E., Miyakawa, A., Jagust, W. & D'Esposito, M. Working memory capacity predicts dopamine synthesis capacity in the human striatum. *J Neurosci* **28**, 1208-1212 (2008). <https://doi.org:10.1523/jneurosci.4475-07.2008>

3 Landau, S. M., Lal, R., O'Neil, J. P., Baker, S. & Jagust, W. J. Striatal Dopamine and Working Memory. *Cerebral Cortex* **19**, 445-454 (2009). <https://doi.org:10.1093/cercor/bhn095>

4 Schuster, B. A. *et al.* Dopaminergic Modulation of Dynamic Emotion Perception. *The Journal of Neuroscience* **42**, 4394 (2022). <https://doi.org:10.1523/JNEUROSCI.2364-21.2022>

5 Kimberg, D. Y., D'Esposito, M. & Farah, M. J. Effects of bromocriptine on human subjects depend on working memory capacity. *NeuroReport* **8** (1997).

6 Mattay, V. S. *et al.* Effects of Dextroamphetamine on Cognitive Performance and Cortical Activation. *NeuroImage* **12**, 268-275 (2000). <https://doi.org:10.1006/nimg.2000.0610>

7 Gibbs, S. E. & D'Esposito, M. Individual capacity differences predict working memory performance and prefrontal activity following dopamine receptor stimulation. *Cogn Affect Behav Neurosci* **5**, 212-221 (2005). <https://doi.org:10.3758/cabn.5.2.212>

8 Frank, M. J. & O'Reilly, R. C. A mechanistic account of striatal dopamine function in human cognition: psychopharmacological studies with cabergoline and haloperidol. *Behav Neurosci* **120**, 497-517 (2006). <https://doi.org:10.1037/0735-7044.120.3.497>

9 Rostami Kandroodi, M. *et al.* Effects of methylphenidate on reinforcement learning depend on working memory capacity. *Psychopharmacology* **238**, 3569-3584 (2021). <https://doi.org:10.1007/s00213-021-05974-w>
