# Supplementary material for: Disruption of dopamine D2/D3 system function impairs the human ability to understand the mental states of other people
Source: PLoS Biol. 2024 Jun 13;22(6):e3002652. doi: 10.1371/journal.pbio.3002652 (PMC11175582; doi:10.1371/journal.pbio.3002652)
Supplement: S2 Results — (DOCX) [file pbio.3002652.s010.docx]

**Dopamine challenge reduced walking speed in individuals with low estimated dopamine synthesis capacity^[[1]](#footnote-1)^**

A Bayesian mixed effects model (Model 7) of drug (PLA, HAL; dummy coded) and WM group (low, high; effects coded) predicting walking speed revealed a negative main effect of drug (${E\mu}_{PLAvsHAL}$ = -0.04, CrI = [-0.08, 0.01], P(${E\mu}_{PLAvsHAL}$) < 0 = 0.95), indicating that, overall, haloperidol tended to reduce walking speed. In addition, there was a main effect of WM group (${E\mu}_{lowWM}$ = -0.04, CrI = [-0.09, 0.01], ${E\mu}_{highWM}$ = 0.04, CrI = [-0.01, 0.09]), demonstrating that under placebo, the low WM group exhibited a slower walking pace relative to high WM individuals (low WM: mean [M] = 1.05 m/s, high WM: M = 1.13 m/s). There further was an interaction between drug and WM group (${E\mu}_{PLAvsHAL,lowWM}$ = -0.04, CrI = [-0.09, 0.00], ${E\mu}_{PLAvsHAL,highWM}$ = 0.04, CrI = [0.00, 0.09],). Separate post-hoc models for low and high WM groups indicated that, whereas the drug slowed movement speed in the low WM group (Model 7.2: (${E\mu}_{PLAvsHAL}$ = -0.08, CrI = [-0.16, -0.01], $P({E\mu}_{PLAvsHAL}<1)$ = 0.99), there were no drug effects on movement in the high WM group (Model 7.3: (${E\mu}_{PLAvsHAL}$ = 0.00, CrI = [-0.04, 0.06]).

1. Results published in our previous paper: Dopaminergic Modulation of Dynamic Emotion Perception (Schuster et al., 2022, *JNeurosci)*. [↑](#footnote-ref-1)
