# Supplementary material for: Disruption of dopamine D2/D3 system function impairs the human ability to understand the mental states of other people
Source: PLoS Biol. 2024 Jun 13;22(6):e3002652. doi: 10.1371/journal.pbio.3002652 (PMC11175582; doi:10.1371/journal.pbio.3002652)
Supplement: Attachment — Submitted filename: response to reviewers.docx [file pbio.3002652.s011.docx]

**Reviewers' comments and author responses**

**Reviewer #1**: This was an well-written, clear, and interesting paper reporting the effects of a D2 DA challenge on theory of mind performance in healthy adults. I am not an expert in either pharmacology nor kinematic analysis, though, I do know about much of the work that has attempted (with varying degrees of success) to connect DA functioning more broadly with theory of mind.

The authors use a relatively novel task within a convincing design (double-blind placebo) to show that DA challenge affects labeling of animations that require mentalizing. The DA challenge also affects the labeling of animations that don't require mentalizing. The effect of DA challenge on mentalizing on non-mentalizing was not statistically distinguishable in the main task. Yet they conclude that DA has a specific effect on mentalizing. This conclusion is based on subsidiary analyses that I didn't think provided empirical support commensurate with the strength of the conclusions.

We would like to thank the reviewer for their input. We agree that these claims should be toned down given the strength of evidence. We have revised the Discussion accordingly, with a particular emphasis on urging caution in interpreting the Results of our subsidiary analysis:

Lines 453-455: “*We thus speculate that these results suggest a common dopamine modulated mechanism among mental state attribution and emotion recognition ability that is independent of working memory function as indexed by the Sternberg^1^ visual WM task.”*

Lines 522-526: *“Second, our interpretation that the observed effects of our dopaminergic manipulation in the mental- and non-mental state conditions arise from two separable neurochemical (i.e., mesolimbic and nigrostriatal) and mechanistic (action representation and reward processing) pathways is based on subsidiary analyses and warrants further investigation using dedicated experimental designs.”*

It was unclear to me what the kinematic analyses showed with respect to the main research question — the authors previously showed that the similarity that one's own movements show to a model's movements predict their accuracy on the mentalizing animations. This effect goes away where in the DA challenge, but they speculate that this is because movements under DA challenge are odd for participants. They show that movement similarity under placebo predicts mentalizing accuracy under DA challenge, but I struggled to see how this shed light on the connection between DA and animation labeling accuracy. I gather that the authors are trying to argue that even though DA affects movement, it's not the effects on movement that are directly affecting their ToM accuracy. But this seems like argument from null and is pretty weak.

The kinematic analysis was included to test hypothesis 2 wherein we predicted that dopamine disruption would affect the extent to which individuals make use of their own motor codes when judging the observed movements in the animations task (see Introduction line 49 and following). This hypothesis is motivated by evidence that one’s own actions support the perception of others’ actions (ranging from simple action understanding^2^ to action-based emotion recognition^3^, intention attribution^4^, and mental state attribution^5^); as well as a considerable literature that strongly supports dopaminergic contributions to motor control^6-9^. Results of Model 2.1 (line 291 and following) revealed that there was an interaction between drug, jerk difference and mental state such that the relationship between jerk difference and accuracy that we observed under placebo was diminished under haloperidol. However, the Reviewer is correct that this result is not strong, independent evidence that haloperidol’s effects on judgements are mediated by effects on movement because the result could be interpreted in various ways. For example, it could be that participants rely less on their own motor codes (perhaps relying more on other sources of information such as visual codes) when judging animations under HAL. Alternatively, participants may rely on their own motor codes to an equal extent under HAL and PLA, but under HAL they are relying on stored motor codes acquired across the lifetime^10,11^ (i.e., their placebo movements), rather than those modified codes (i.e., modified by HAL) via which they are currently performing action.

We implemented Model 3 to test this possibility and found evidence to support the latter interpretation. Model 3 revealed that when participants were labelling animations under haloperidol, accuracy in those judgements was affected by their own movements produced in the placebo condition. Note that Model 3 affords us the opportunity to argue from a positive, not a null, result. The combination of kinematic analyses (Model 2.1 and Model 3) shows that under both HAL and PLA participants’ accuracy on the animations task is influenced by the similarity of the animation to the movements that they produce in the placebo condition. Thus, suggesting that stored motor codes acquired across the lifetime, and upon which sensorimotor internal models are fine-tuned^10,11^, contribute to animations task accuracy. We have now added additional clarification to the manuscript (see below) and thank the Reviewer for helping us to clarify the contribution of this analysis.

Lines 314-337: *“This result affords various interpretations. For example, it could be that participants rely less on their own motor codes (perhaps relying more on other sources of information such as visual codes) when judging animations under HAL. Alternatively, participants may rely on their own motor codes to an equal extent under HAL and PLA, but under HAL they are relying on stored motor codes acquired across the lifetime, and upon which sensorimotor internal models are fine-tuned^10,11^ (i.e., their placebo movements), rather than those modified codes via which they are currently performing action.”*

Lines 350-352: *“Models 2.1 and 3 show that under both HAL and PLA participants’ accuracy on the animations task is influenced by the similarity of the animation to the movements that they produce in the placebo condition.”*

More interesting was that DA challenge also affected emotion recognition accuracy in a separate task, and the extent to which it did so for a given participant was weakly correlated with their decrement in performance on the mentalizing animations. The authors speculate that this is evidence for a pathway whereby DA affects social cognition independent of any effects that it might have on other aspects of cognition —  they single out executive function as an important example. Perhaps I don't know enough about the movement measures, but since EF is not measured here, it's not clear how this speculation is supported. Do we know that EF is not important for the emotion recognition task used here (I liked, but was new to me), or just not for other emotion recognition tasks generally?

We thank the reviewer for this thoughtful comment. Our speculation that the observed relationship between drug effects on mental state attribution and emotion recognition stem from a pathway which does not rely on executive functions arises from the fact that in this same model, drug effects on working memory function did not contribute to predicting changes in mental state attribution accuracy. That is, we were using the working memory task as a proxy measure of executive function. Intriguingly, we previously found that drug effects on the emotion recognition task were related to drug effects on working memory function^12^. Thus, our results from the current and our previous study^12^ suggest that the drug-induced variance shared between emotion recognition and working memory function does not overlap with the drug-related variance shared between emotion recognition and mental state attribution.

Prompted by another reviewers’ comments, we rephrased the following section in the Discussion, to acknowledge that working memory function does not represent the entirety of executive functions, and therefore it is still possible that other executive processes such as attention or inhibitory control play a role in the observed effects:

Lines 528-531: *“Third, while our results suggest that haloperidol affected mentalising performance independent of working memory function, this does not preclude other aspects of executive function, such as attention or inhibitory control, playing a role in the dopaminergic modulation of mentalising.”*

In general, I found these data very interesting, but the strong conclusions not yet warranted.

A minor consideration — there is a small amount of evidence that DA might be related to some aspects of children's theory of mind development (e.g., Lackner et al., 2010; Sabbagh et al., 2012), also independent of whatever effects DA might have on EF. Also, the Savina & Beninger paper comparing the effects of typical and atypical antipsychotics (cited near the end of this paper) might provide interesting evidence — taking haloperidol didn't increase ToM performance in this group, but taking clozapine and olanzapine did (without affecting control tasks). I don't know all the pharmacology here (except that I know it's complicated), but it seems like a dissociation worth mentioning in the intro trying to understand current state of the evidence on DA and ToM.

We thank the reviewer for this thoughtful addition and have reflected upon this in the Introduction:

Lines 27-32: *“Evidence from studies examining those with schizophrenia is equally inconclusive: For instance, one study^13^ reported improvements in ToM ability in patients treated with certain atypical antipsychotics (e.g., olanzapine), and detrimental effects of typical antipsychotic medication (e.g., haloperidol), but putative selection bias and the lack of on-off comparisons make it impossible to clearly attribute group differences to effects of the dopaminergic drugs.”*

Please note that due to the on-going debate about the reliability of studies using the eye-blink method to index dopamine function (see ^14,15^) we have chosen not to include studies using this method.

**Reviewer #2**: This is an interesting study of the effects of a dopamine antagonist on the attribution of intention to moving shapes in healthy adults. The paper makes a case for a causal implication between dopamine and the theory of mind, and that dopaminergic modulation of mental and non-mental state attributions involve distinct pathways.

There are clear strengths in the within-subject design, placebo control, and analysis. The links with motor responses were an interesting hypothesis.

My three primary concerns are that the mental and non-mental state categorisation does not seem to be meaningful, dependent variables were used inconsistently, and a single working memory task is insufficient to rule out general effects on executive function and task performance, as claimed. In the least charitable interpretation, we see that haloperidol reduces computerised task performance, including deducing what might be intended in an animated triangles video and communicating that deduction. In the most charitable interpretation (with potential revisions), we see that a dopamine antagonist, directly or indirectly, may influence the ability to infer mental states, independent of a specific type of working memory.  As discussed below, I would not endorse the exploratory analyses based on video categories at this time.

For consideration:

*	Animation target words were: 'fighting, following, seducing and surprising'.  The experimenters divided these four words into two categories: based on Schuster et al 2021, 'following' and 'fighting' as 'non-mental state' (because there is reciprocal interaction, but supposedly no implication that one character was reading the other triangle's 'mind') and 'seducing' and 'surprising' animations as 'mental state' attributions because one character reacts to the other character's mental state. There is nothing I could find in the paper to justify the classification.

If the label 'mental state' attribution refers to the participant's inference of mental states, then participants could attribute a mental state to 'fighting' and 'following' to at least one, and potentially two triangles in the animation, but these are classified as non-mental state attributions. There is the intentionality of a triangle in each of these concepts, which the participant could infer.

If the label 'mental state' attribution refers to inferring the triangle's inference of mental states, then it is unclear why that would be interesting. Even so, at least one and possibly two triangles are likely to infer the mental state of the other when fighting or following them.

So, what I am trying to say is that an experimental distinction between these categories does not appear to be correct or meaningful.

The specific selection of animation types was based on Schurz et al. (2014) ^16^ (for the autism work see^17^; there are multiple other meta-analyses in developmental populations and specific clinical groups). This work has its roots in philosophy of mind and in the original work on theory of mind within comparative psychology. The approach taken across these hundreds of papers is remarkably consistent and we have adopted the same approach. Specifically, our non-mental and mental state conditions are the goal-directed (G-D) and Theory of Mind (ToM) conditions, respectively, in the seminal study by Abell et al.^11^ (and the studies which followed) who described the intended differences between their conditions as follows: *“In each [goal-directed] sequence there was reciprocal interaction, but no implication that one character was reading the other's ‘mind’.* *The ToM sequences, by contrast, showed one character reacting to the other character's mental state.”* (^11^, p. 5).  The key distinction between the mental state and non-mental state words is that the mental state words can be unpacked as propositional attitudes of this form “I intend to cause a particular mental state in the other”. That is, seducing can be unpacked as “I intend to cause the other to be aroused” and surprising can be unpacked as “I intend to cause the other to be surprised” (see ^18^). Following and fighting do not describe situations in which one triangle intends to cause a particular *mental state* in the other. Even if they are described in a maximally mentalistic fashion they still only describe a situation in which one triangle intends to modify their movements, in a particular way, in relation to the movements of the other triangle but with no aim of causing a particular mental state in the other.

The distinction between our mental and non-mental state conditions is corroborated by empirical work illustrating that ToM animations consistently elicit higher attributions of intentionality relative to goal-directed animations in non-autistic participants^13-15^.  Furthermore, clinical studies show differences between autistic and non-autistic participants^10-14^, as well as relationships between autistic traits and accuracy, for ToM, but not goal-directed animations^12^.

The Reviewer is absolutely correct in that … “participants could attribute a mental state to 'fighting' and 'following'” … The difference we draw, which is mirrored in the previous studies in the field, is that the two categories require different degrees of mentalising (e.g., the mental state videos are intended to represent an intention in one agent to *cause* a particular mental state in the other). Supporting this, a recent fMRI study showed parametric modulation of activation in ToM-associated areas by animation types intended to pose increasing mentalising demands (random movement > goal-directed > ToM)^18^.

We have updated our motivation for the choice of animations used in our study accordingly:

Lines 141-149: “*Within each mental state category, two words describing the interactions were chosen in equivalence to the words used in a seminal study by Abell et al.^19^ and multiple following studies^20-23^: Mental state: seducing, surprising; non-mental state: following, fighting. The key distinction between the two conditions is that the mental-state (ToM) animations entail propositional attitudes wherein one agent intends to cause or act upon a particular mental state in the other, while non-mental state (goal-directed) animations do not require such causal inference^18^. This is corroborated by prior research where the latter have been shown to consistently elicit lower levels of spontaneous attributions of intentionality than the mental state animations^20-22^.”*

Without a detailed reading of the methods and a glance at Schuster et al. 2021, a reader could have assumed that 'non-mental state' attribution referred to a variant of the animation task where they moved randomly or in geometric patterns, which would perhaps have been more meaningful.  So as a more minor point, the distinction between the conditions should be clearly communicated in the abstract, intro and discussion.  These categories are not considered in the introduction at all.

We thank the reviewer for this suggestion and agree that the specific nature of our non-mental state condition could have been communicated more clearly in parts of our manuscript. We have revised the manuscript accordingly, added descriptions to the Abstract and Introduction, and extended our existing description of the task in the Methods section.

Abstract: *“[…] videos of two triangles depicting mental state (involving mentalistic interaction wherein one triangle intends to cause or act upon a particular mental state in the other, e.g., surprising) and non-mental state (involving reciprocal interaction without the intention to cause/act upon the other triangle’s mental state, e.g., following) interactions.”*

Introduction (lines 69-73): *“Thus, using our own previously developed^5^ version of a well-established mentalising task – the animations task, wherein participants label videos depicting mental (e.g., seducing) and non-mental (e.g., following) states – here we first employed a pharmacological dopamine manipulation in healthy volunteers to investigate whether disruption of dopamine system function plays a causal role in mentalising.”*

Methods (lines 135-141): *“Animations tasks typically involve participants viewing and interpreting short videos of interacting triangles which have been animated to either display so-called mental state interactions (i.e., mentalistic interaction wherein one triangle intends to cause or act upon a particular mental state in the other e.g., ‘surprising’; in prior research referred to as ‘ToM’ interactions) or non-mental state interactions (also called ‘goal-directed’ interactions; involving reciprocal interaction without the intention to cause/act upon the other triangle’s mental state, such as ‘following’).“*

*	Unfortunately, I do not agree with the experimenter's conclusion that these results suggest a common dopamine-modulated mechanism among mental state attribution and emotion recognition ability independent of executive function. A single working memory task is insufficient to measure executive function to make any inferences beyond that type of working memory. This is because executive function also involves response inhibition, interference control (e.g., selective attention), and cognitive flexibility. Diamond A. Executive functions. Annu Rev Psychol. 2013;64:135-68.

We thank the reviewer for this valuable point. Upon reflection we agree that our results do not imply that the dopaminergic modulation of mental state attribution was independent of any aspect relating to executive functions. We have revised the Discussion to better reflect this, using the reference the reviewer kindly provided:

Lines 528-531: “*Third, while our results suggest that haloperidol affected mentalising performance independent of working memory function, this does not preclude other aspects of executive function, such as attention, inhibitory control, or cognitive flexibility^24^ playing a role in the dopaminergic modulation of mentalising.”*

*	Line 324. It is unclear why the inability to compare PLA and HAL trials directly, due to conditions not experiencing the same videos, led to a choice for a very noisy binary measure of accuracy on the triangles task and a new measure of 'percentage accuracy'.  With such a noisy measure, a trial in which the target word was rated 99% higher is weighted the same as a trial where it was rated 1% higher than the others.  So that is not preferable and it is unclear how this solves the incomparability problem between conditions. In the main analysis, a random intercept was introduced for the animation ID. Why not do that here as well?  The disconnect between this exploratory analysis and the main analysis is somewhat troubling and requires further explanation.

We thank the reviewer for highlighting areas where we could more clearly explain the reasoning behind our calculation of drug-induced changes in animation task performance. In each drug condition (placebo vs. haloperidol), participants viewed eight different animations for each of the four different words (i.e., 16 animations/trials per mental state condition and drug condition). Although animations belonging to the same word category (e.g., surprising) can be classified according to several shared similarities (e.g., kinematics, see Schuster et al., 2021), we assume that the between-animation variance within one word category is non-negligible. Therefore, since the animations viewed on the placebo and haloperidol day are different (even when they relate to the same word category), no meaningful trial-wise calculation of performance change between the two drug categories was possible (any selection of pairs of trials to compare between drug conditions would have been completely arbitrary, therefore inducing unwanted random variance). For instance, for the word surprising, a participant may have viewed and rated animations with the following IDs, in the following order (for ease different IDs are denoted by different letters): [haloperidol: B, A, H, E, Z, K, I, L; placebo: H, M, I, C, R, D, A, W]. In this example, calculation of HAL-PLA differences for one and the same animation would not have been possible for animations with the IDs B, E, Z, K, L, M, C, R, D, W. As we hope is evident from the previous example, a random intercept for animation ID would only have been possible if there had been directly comparable animation pairs with a one-to-one match to a single animation ID.

We therefore chose to re-conceptualise the accuracy measure in terms of a binary hit/miss variable, allowing us to forgo the arbitrary selection of animation pairs (by just comparing, between drug conditions, the overall change in hits and misses within a word category). We agree with the reviewer that this measure is relatively crude, however we could not conceive of any more sensitive calculation of drug effects in the animation task.

We highlight our motivation for the binary measure in the Results section:

“Due to the random selection of animations (see ^5^), participants did not necessarily view the same animations in PLA and HAL trials, making it impossible to calculate a trial-by-trial measure of drug related changes.” (lines 360-362).

It may provide some reassurance that a binary variable for accuracy in the main analysis (calculated the same way as reported for the secondary analyses), albeit noisy, provides sufficient sensitivity to produce results comparable to those reported for the original model 1.2 (${E\mu}_{HALvsPLA}$ = -0.40, CrI = [-0.74, -0.07]; ${E\mu}_{HALvsPLA, mentalVSnon-mental}$ = 0.05, CrI = [-0.39, 0.47]; ${E\mu}_{mentalVSnon-mental}$ = -1.44, CrI = [-1.97, -0.93]).

*	I worry that many of the credibility intervals overlap with zero (all the parameters for model 4.2 in Supp Table 4.3). Does this support the concrete inferences the authors are making? Figure 3 suggests there is not really a strong delineation between mental state attribution categories and correspondence between triangles task performance and emotion recognition performance. The reported stats written in the main text for model 4.2 do not match the stats reported in Supp Table 4.3, which does not reassure that model fitting was done correctly and selectively. I am not commenting further on the Bayesian stats, because they are beyond my expertise, so I hope another reviewer may offer their thoughts on them. They should have specialist (which I am not) reviewer attention before acceptance for publication.

We understand the Reviewer’s concerns, which we presume are informed by a frequentist perspective, about the credible Intervals containing zero. From a Bayesian perspective, however, although the credible intervals for model 4.2 include zero they are strongly skewed, meaning they are not *centered* around zero and therefore still represent meaningful effects (i.e., effects that are informative and useful in guiding future research). The fact that the intervals include zero simply reflects that there is *some* uncertainty surrounding the effect relative to the chosen credible interval. Importantly, and as illustrated by Bayesian Statisticians Richard McElreath^25^ (e.g., pp. 56, 58) and Andrew Gelman (https://statmodeling.stat.columbia.edu/2016/11/05/why-i-prefer-50-to-95-intervals/), as Bayesian analyses are not bound by thresholds at which to accept or reject a null hypothesis, they are not bound to a specific credibility interval either. The choice of the specific interval of certainty is arbitrary, and the most important thing is to express the uncertainty in the posterior in a clearly specified way. If we had used 89% credible Intervals instead of 95%, as some Bayesian researchers like McElreath have been doing to emphasise the arbitrariness of such values, these would have excluded zero. Most importantly, this increased uncertainty relative to our chosen 95% Intervals only concerns our control and secondary analyses. To be fully transparent we now have clearly flagged in the manuscript the cases where this applies:

Lines 267-275: *“There was no interaction between drug and drug day, indicated by a negative effect of drug for drug day 1 (EμHALvsPLA,day1 = -0.52, CrI = [-1.07, 0.03]; note there is slightly increased uncertainty around the drug effect in this model, as shown by the CrI including zero), which did not differ from the drug effect on day 2 (EμHALvsPLA, day1vsday2 = -0.06, CrI = [-0.81, 0.69]). Model 1.4 was fit to drug and arousal (participant reported tiredness levels ranging from 1 = not tired at all to 10 = maximally tired; collected before the main task) predicting accuracy. The model revealed a preserved effect for drug ([Equation] = -0.53, CrI = [-1.13, 0.06], again with minimal increase in uncertainty surrounding the effect) [...]”*

Lines 400-420: *“[…] and, relative to non-mental state animations, a small effect indicating a positive relationship between emotion recognition change and animations task accuracy change for mental state animations (*${E\mu}_{ERchange, mentalVSnon-mental}$ *= 0.06, CrI = [-0.01, 0.13];* $P({E\mu}_{ERchange, mentalVSnon-mental}>0)$ *= 0.94; coefficient for mental state animations* ${E\mu}_{ERchange,mental}$*: -0.01 + 0.06 = 0.05; note the CrI including zero indicates some uncertainty surrounding this effect).”*

We thank the reviewer for pointing out the discrepancy between the values reported in the manuscript and in the Supplementary Materials. To explain the reason for this discrepancy: the statistics in our manuscript are derived from *systematic random sampling methods* (MCMC sampling), resulting in small differences between model parameters if a model is re-run without setting a specific seed (used by the random number generator). The statistics reported in the Supplementary Materials align with the ones produced by the reproducible R Markdown script at <https://osf.io/xm7ty/>, as here the same seed was used. We have now updated the values in the manuscript to match with the ones reported in the Supplementary Materials.

Minor comments:

*	Line 328 There are four words in each drug condition, so how do you end up with four percentage accuracy scores and not 8? Presumably, they developed percentage accuracy scores per category…..

We thank the reviewer for alerting us to a discrepancy in this description. There were actually only *two* – not four – percentage accuracy values per participant/word/drug condition, summing up to *eight* percentage accuracy values per participant in total (1 percentage accuracy value for each of the four words surprising, seducing, following, fighting = 4 x 2 drug conditions = 8). We have updated this in the manuscript:

Lines 365-367: *“Subsequently, the percentage of correct trials out of all eight trials for a given word was calculated, resulting in two percentage accuracy values per animation word per participant (1 PLA, 1 HAL).”*

*	Model results, in tables, may be more appropriate in the main text.

As this manuscript reports a large number of models, we believe reporting the data in tables would break up the running text and interfere with readability. We have therefore chosen to display the model results in the main text, in line with other comparable research papers recently published in PLoS Biology (e.g., ^26-28^).

*	For the modelling (Line 376), it seems that random intercepts (subject IDs) may have not been included after deciding that they did not contribute to model fit. I would suggest this should be included regardless, as a minimum.

We thank the reviewer for this comment. We understand why one might suggest to keep the random effects in the model despite the fact that model comparison shows random between-participant variance does not meaningfully contribute to model predictive strength. However, the transformation of the outcome variable to difference values already removed all random between-participant variation. This is because, for each subject, any individual tendencies that would have led to between-participant variation are similar across the tasks. Thus, including the random effects in this case is redundant.

*	Line 395: Why are the units of increase in emotion recognition standard deviations, but units of increase of triangles accuracy in %? Why not standardise both? What does '5% increase in their ability' mean when the outcome is a binary variable?

We thank the reviewer for raising this issue with Model 4.3. and understand why our interpretation of the coefficient in terms of % may have caused confusion. The outcome variable of this model was calculated from binary scores, but is itself ordinal, and represents percentage change: Animations task accuracy change values range from -1 (i.e., PLA: 0 out of 8 animations correctly identified, HAL: 8 out of 8 correctly answered) to + 1 (PLA: 8 out of 8 correct, HAL: 0 out of 8 correct). The 5% increase referred to the total of 8 animations, which means that an increase in 1SD in emotion recognition performance under HAL was associated with participants correctly identifying roughly half a trial more (0.05 * 8 = 0.4), relative to those participants who showed no change in emotion recognition performance. Accordingly, an increase of 2 SD in emotion recognition performance was associated with participants correctly identifying about 1 animation more than under placebo (0.05 * 2 * 8 = 0.4). We have amended the relevant section in the results to better illustrate this accordingly:

Lines 420 – 423: *“Thus, for every 2 SD increase in emotion recognition accuracy after haloperidol, individuals correctly identified roughly one animation more (0.05 * 2 * 8 = 0.8) than under placebo, relative to those participants who showed no change in emotion recognition performance.”*

Considering the Reviewer’s question referring to standardising both variables: The emotion recognition change variable was standardised to allow comparison with other predictors (i.e., WM change) that were previously in the model. As for the outcome variable percentage accuracy change: This variable – even though it was treated like a continuous variable after model comparison favoured the continuous approach – is ordinal and therefore arithmetic operations like z-standardisation are meaningless (i.e., there are no meaningful arithmetic mean and standard deviation).

*	Line 423-426: The author's phrasing implies a double dissociation, but it should be clear that this is not the case and that inferences should not be made from a negative result.

We thank the Reviewer for highlighting this. We have checked through the document to ensure that we do not imply a double dissociation in any part of the manuscript.

*	The 'causal 'relation claim has been overstated, which could mistakenly be used as a cornerstone of a theory that dopamine directly modulates mentalising.  The authors should be very clear that it is possible that haloperidol could have influenced task performance based on other cognition (such as attention) and other neurotransmitter systems.  Further tests in mentalising tasks with different cognitive demands, would have been more supportive of the claim.

We thank the Reviewer for drawing our attention to this. We now added a new paragraph to further highlight that haloperidol could have led to the observed decreases in (mental) state attributions via modulating general cognitive functions, such as attention or inhibitory processes:

Lines 528-531: “*Third, while our results suggest that haloperidol affected mentalising performance independent of working memory function, this does not preclude other aspects of executive function, such as attention, inhibitory control, or cognitive flexibility^24^ playing a role in the dopaminergic modulation of mentalising.”*

We have also modified the opening paragraph of the Discussion such that we emphasise the indirect nature of the effects of haloperidol:

*“Our findings thus show that dopaminergic pathways are, at least indirectly, involved in Theory of Mind.”* (Lines 437-439)

Furthermore, we would like to highlight the limitations section of the Discussion, in which we have flagged that interactions with other neurotransmitter systems may have additionally affected our results:

Lines 531-537: *“Finally, dopamine is likely not acting on social function in isolation. There is growing evidence that the dopaminergic system may work in interaction with the serotonergic system, evidenced for instance by reports of therapeutic effects of atypical (acting on both dopaminergic and serotonergic receptors), but not classical (targeting specifically dopamine receptors), antipsychotics on ToM^13,29,30^”*

*	There does not seem to be any consideration of haloperidol's side effects.

We thank the reviewer for this highlighting this. Side effects in our study were rare and non-serious, with only three of 43 participants reporting mild adverse effects (fatigue, mild headache) after receiving the drug. Out of these three participants, only two are included in the dataset reported here (due to COVID-19 related discontinuation of the study for one participant). More importantly, two participants reported the same kind of side effects after having received the placebo, supporting the idea that the reported symptoms in all cases could have been attributed to drug-unrelated factors such as computer eye strain. We therefore think that the impact of side effects on our results is negligible. We have added this relevant information to the Methods section:

Lines 110:113: *“Participant reported adverse responses were rare (five out of 43 participants) and generally mild, with the most frequent symptoms mentioned being fatigue and headache. Importantly, only three of five participants reported side effects after having received haloperidol.”*

i wish you the very best of luck with this interesting line of work.

**Reviewer #3**: I would like to thank the authors for their fascinating paper. The authors use a D2 dopamine antagonist challenge to test the role of dopamine transmission on mental and non-mental state inferences in healthy humans adults. Authors found that haloperdol reduced inferences across the board with no special case for mental state inferences, and that D2 dopamine antagonism also broke the relationship between kinematic similarity and accuracy. I would highly recommend the publication of this article, although there are a few minor comments I had which I feel would strengthen the manuscript and make the language clearer.

Introduction:

- The authors have missed out some key (albeit recent) literature on the causal role of dopamine in social cognition and theory of mind

Use of D2 antagonism to examine trust in humans: 1. Mikus, N., Eisenegger, C., Mathys, C., Clark, L., Müller, U., Robbins, T. W., ... & Naef, M. (2023). Blocking D2/D3 dopamine receptors in male participants increases volatility of beliefs when learning to trust others. Nature Communications, 14(1), 4049.

Use of D2 antagonism to examine intentional mental state inferences in humans: 2. Barnby, J. M., Bell, V., Deeley, Q., & Mehta, M. A. (2020). Dopamine manipulations modulate paranoid social inferences in healthy people. Translational psychiatry, 10(1), 214.

Use of rat models to examine social interaction: 3. Homberg, J. R., Olivier, J. D., VandenBroeke, M., Youn, J., Ellenbroek, A. K., Karel, P., ... & Ellenbroek, B. A. (2016). The role of the dopamine D1 receptor in social cognition: studies using a novel genetic rat model. Disease models & mechanisms, 9(10), 1147-1158.

We would like to thank the reviewer for these excellent suggestions. We had already included a reference to the first article in the Introduction but have now updated this from the preprint to the article reference, and have additionally added a reference to the second article:

Lines 39-42: *“While a handful of existing studies show effects of dopamine D2 receptor antagonism on emotion recognition^12,31^ and social belief updates^32,33^, to the best of our knowledge, no study to date has explored effects of dopamine manipulation on ToM function in healthy individuals.”*

We also included the Barnby et al. paper in the Discussion as follows:

Lines 460-468: *“Alternatively, recent research suggests that haloperidol may have affected the processing of social cues via coding for their perceived self-relevance: For instance, in a simple dictator game, haloperidol resulted in a shift in intentional attributions to a partner’s behaviour along an axis of self-relevance, leading to a reduction in attributions of harmful intent (i.e., relevant to the participant as harmful intent represents threat) alongside an increase in attributions of self-interest (not relevant to the participant)^32^. While self-relevance has been shown to impact stimulus processing at various stages (e.g., visual, attention, memory^34,35^), it is yet unclear whether self-relevance and reward effects rely on shared or distinct mechanisms^36^.”*

After careful consideration we decided not to include suggested article 3, as D1 and D2 dopamine action are thought to have opposing effects on neural function^37^ and thus a meaningful discussion would have gone beyond the scope of our current manuscript.

- The examination of mentalising in the task you use is specific to a class of mentalising that does not require representation, only inference (Conway et al., 2019; Psych. Bulletin & Review). Importantly, the class of inference used is specifically observational, as the outcomes of the interaction do not involve joint rewards for both the player and the observed agent (in this case, non human shapes). I think this needs to be made clearer in the introduction OR discussion with respect to the limitations of the task. It may well be that D2 dopamine disrupts mental state _representation_ given its role in model-based reasoning (Mikus et al., 2022; elife).

Thank you for highlighting this important point. Ongoing discussions about representation vs. inference in ToM are of great interest. Conway et al. 2019, for example, argue that … ‘on any particular test one could make an inaccurate mental state inference yet still represent a mental state. In such a situation there is no deficit in the representation of mental states, but rather a deficit in accurately inferring the content of a particular mental state’… This work highlights the possibility that under HAL and PLA participants are equally proficient in *representing* mental states, however, under HAL participants are less accurate in *inferring* the contents of a particular mental state. To be able to make such inferences further work would need to use paradigms that are specifically designed to isolate the representation and inference of mental states. We have added to the limitations section a sentence where we consider our results in relation to ongoing discussions about representation vs. inference in ToM:

Lines 518 – 522: *“Moreover, while previous work suggests that dopamine may modulate mental state representation (given its role in model-based reasoning^38^), based on the current data it cannot be determined whether performance differences are driven by differences in the representation, or mere inference of those mental states^39^.”*

Methods:

- Were alcohol use, smoking or other drug use an inclusion criteria for the participants? Was urinalysis conducted to eliminate the possibility of concomitant drug use?

- Was arousal over the active drug period monitored? e.g. feeling dysphoric, lethargic, or uncomfortable?

Our study followed standard procedures for psychopharmacology studies. This included using alcohol/drug dependency or recent (within last 7 days) recreational drug use as exclusion criteria for the study. We have added the complete list of inclusion/exclusion criteria to the Supplementary Materials (Appendix 3). Urinalysis was not conducted as it is not standard at our institute to do so and is prohibitively expensing in terms of time and budget.

Arousal was monitored across the whole study day as self-reported tiredness on a scale from 1 (not tired at all) to 10 (maximally tired). We have added a control analysis (Model 1.4) confirming that it was not the drug’s effects on arousal that resulted in the observed performance effects in the main task:

Lines 271-279: *“Model 1.4 was fit to drug and arousal (participant reported tiredness levels ranging from 1 = not tired at all to 10 = maximally tired; collected before the main task) predicting accuracy. The model revealed a preserved effect for drug (*${E\mu}_{HALvsPLA}$ *= -0.53, CrI = [-1.13, 0.06]) and no main effects for arousal (see Supplementary Materials). There was an interaction between drug and a 7^th^ order polynomial trend for arousal, however model comparison between models 1.1 and 1.4 confirmed that arousal level did not meaningfully contribute to explaining variance in accuracy; we therefore did not interpret this result any further.”*

- In the animations task, why is fighting considered a non-mental state inference? If I was fighting someone the inference would be that I was angry or frustrated in some way. Likewise, at a push, we could consider following social given the inference that the other shape was worth following, or even that there was some group cohesiveness that may be at play? This is not to say the task isn't appropriate, but some clarity around the reasoning of the delineation between mental state and non-mental state would be useful as it was not clear to me. This also has an effect on the results, as if they're all somewhat mental state inferences it skews the interpretation.

We thank the reviewer for highlighting that the conceptual distinction between our mental and non-mental state conditions and the choice for the individual words within each condition could have been communicated more clearly. Please see above (pages 4-5) for our response to a similar concern to Reviewer 2.

Results:

The analyses were well conducted and reported clearly.

Discussion:

- In the last sentence of the first paragraph, given the previous studies listed above in my comments during the introduction, I would argue that this study also contradicts the other previous findings with respect to Theory of Mind, given that Theory of Mind is a term roughly identified as both inference AND representation of social others. At the moment I think the statement is a little misleading. There is more evidence than that presented that dopamine is involved in Theory of Mind, mentalising, mental state attribution, and social cognition.

We thank the Reviewer for this observation. We have now amended the sentence such that it reads:

Line 437: “*Our findings thus show that dopaminergic pathways are, at least indirectly, involved in Theory of Mind*.”

We have further added to the limitations section a sentence where we consider our results in relation to ongoing discussions about the difference between representation and inference in ToM (see above).

- Given the clarity as it stands around what counts as a mental state and non-mental state attribution, I am also unclear how to interpret these results. Therefore the first sentence of the second paragraph feels confusing.

- Given the role of D2 antagonism on emotion recognition, it may be that the tasks use only probe specific facets of mentalising/theory of mind, and more interactive tasks (as in Mikus et al., 2022; Barnby et al., 2020) may be testing different components. I would suggets the language around mentalising in this paper be specified to social recognition or social perception. It does not involve the need to represent another social agent (as in repeated, or joint inference social tasks).

- The limitations should outline the drawbacks of the animation task, specifically that it likly does not represent truely social contexts or the need to represent other social agents (see Schilbach 2014, Frontiers in Human Neuroscience).

We hope that the clarity we have provided in the manuscript and our responses to the Reviewer’s above comment regarding the distinction of mental- and non-mental state conditions has resolved their query about the first sentence of the second paragraph.

We agree with the reviewer that, although animations tasks offer certain unique qualities (e.g., experimental isolation of social stimulus processing from face/identity perception as well as the control of task demands to verbal ability), they have clear limitations with respect to ecological validity. We have added the following section to the Discussion to reflect this:

Lines 509-518: *“First, while animations tasks have been widely used to probe socio-cognitive processes due to their ability to reliably distinguish between clinical and control groups^5^, it can be argued that their ecological validity is limited compared to experimental designs involving realistic social interactions. Real-world social situations give rise to a multitude of clues to others’ mental states, including facial expressions, tone of voice and content of verbalisations, and involve repeated interactions - all of which the present task is not designed to index. While the dopaminergic modulation of mentalising during recursive social interactions has been investigated for instance using multi-round economic games^32,33^, future work could expand on the existing results by investigating how dopamine modulates the attribution of mental states to human agents in face-to-face interactions.”*

- The authors cite Mizrahi et al., 2007 as evidence for where dopaminergic treatment leads way to social cognitive dysfunction, although that study actually found that treatment _improved_ social cognitive dysfunction. I am not sure this is the study the authors would want to use for this point, and also contradicts their claim that D2 dopamine dysrupts social cognitive processes. It may be that D2 dopamine is acting on a system with an inverted 'U' optimal transmission window, where too much or too little transmission severely reduces social perception.

We apologise for any confusion regarding our choice of reference for our final statement. Leading on from our current finding that dopamine causally impacts mental state attribution, we intended to use the reference of Mizrahi et al. to emphasise that dopaminergic treatment may help *improve* socio-cognitive abilities in those with imbalances in the dopaminergic system. As the reviewer implied, there is a growing body of research suggesting dopamine may modulate cognitive function via an inverted U function^40^. Accordingly, in the study by Mizrahi et al., dopamine antagonism may have led to improvements in ToM performance of their schizophrenic patients by reducing sub-optimally high dopamine action to more optimal levels.

In line with this, we have added the following clarification to the manuscript:

Lines 532-537: “Although dopaminergic treatment may benefit socio-cognitive function (potentially by reducing sub-optimally high dopamine action to more optimal levels), there is growing evidence that the dopaminergic system may work in interaction with the serotonergic system, evidenced for instance by reports of therapeutic effects of atypical (acting on both dopaminergic and serotonergic receptors), but not classical (targeting specifically dopamine receptors), antipsychotics on ToM^13,29,30^.”

I would like to thank the authors once again for this interesting study and look forward to their revision.

- 1	Sternberg, S. High-speed scanning in human memory. *Science* **153**, 652-654, doi:10.1126/science.153.3736.652
- 10.1126/science.153.3736.652. (1966).
- 2	Aglioti, S. M., Cesari, P., Romani, M. & Urgesi, C. Action anticipation and motor resonance in elite basketball players. *Nature Neuroscience* **11**, 1109-1116, doi:10.1038/nn.2182 (2008).
- 3	Edey, R., Yon, D., Cook, J., Dumontheil, I. & Press, C. Our own action kinematics predict the perceived affective states of others. *Journal of Experimental Psychology: Human Perception and Performance* **43**, 1263-1268, doi:10.1037/xhp0000423 (2017).
- 4	De Marco, D., Scalona, E., Bazzini, M. C., Avanzini, P. & Fabbri-Destro, M. Observer-Agent Kinematic Similarity Facilitates Action Intention Decoding. *Scientific Reports* **10**, 2605-2605, doi:10.1038/s41598-020-59176-z (2020).
- 5	Schuster, B. A. *et al.* Kinematics and observer-animator kinematic similarity predict mental state attribution from Heider–Simmel style animations. *Scientific Reports* **11**, 18266, doi:10.1038/s41598-021-97660-2 (2021).
- 6	Niv, Y., Daw, N. D., Joel, D. & Dayan, P. Tonic dopamine: opportunity costs and the control of response vigor. *Psychopharmacology* **191**, 507-520, doi:10.1007/s00213-006-0502-4 (2007).
- 7	Bakhurin, K., Hughes, R. N., Jiang, Q., Fallon, I. P. & Yin, H. H. Force tuning explains changes in phasic dopamine signaling during stimulus-reward learning. *bioRxiv*, 2023.2004.2023.537994, doi:10.1101/2023.04.23.537994 (2023).
- 8	Bova, A. *et al.* Precisely timed dopamine signals establish distinct kinematic representations of skilled movements. *eLife* **9**, e61591, doi:10.7554/eLife.61591 (2020).
- 9	Hughes, R. N. *et al.* Ventral Tegmental Dopamine Neurons Control the Impulse Vector during Motivated Behavior. *Current Biology* **30**, 2681-2694.e2685, doi:<https://doi.org/10.1016/j.cub.2020.05.003> (2020).
- 10	Hunnius, S. & Bekkering, H. What are you doing? How active and observational experience shape infants' action understanding. *Philosophical transactions of the Royal Society of London. Series B, Biological sciences* **369**, 20130490-20130490, doi:10.1098/rstb.2013.0490 (2014).
- 11	Edey, R., Yon, D., Dumontheil, I. & Press, C. Association between action kinematics and emotion perception across adolescence. *Journal of Experimental Psychology: Human Perception and Performance* **46**, 657-666, doi:10.1037/xhp0000737 (2020).
- 12	Schuster, B. A. *et al.* Dopaminergic Modulation of Dynamic Emotion Perception. *The Journal of Neuroscience* **42**, 4394, doi:10.1523/JNEUROSCI.2364-21.2022 (2022).
- 13	Savina, I. & Beninger, R. J. Schizophrenic patients treated with clozapine or olanzapine perform better on theory of mind tasks than those treated with risperidone or typical antipsychotic medications. *Schizophrenia Research* **94**, 128-138 (2007).
- 14	Sescousse, G. *et al.* Spontaneous eye blink rate and dopamine synthesis capacity: preliminary evidence for an absence of positive correlation. *European Journal of Neuroscience* **47**, 1081-1086, doi:<https://doi.org/10.1111/ejn.13895> (2018).
- 15	Dang, L. C. *et al.* Spontaneous Eye Blink Rate (EBR) Is Uncorrelated with Dopamine D2 Receptor Availability and Unmodulated by Dopamine Agonism in Healthy Adults. *eNeuro* **4**, doi:10.1523/eneuro.0211-17.2017 (2017).
- 16	Schurz, M., Radua, J., Aichhorn, M., Richlan, F. & Perner, J. Fractionating theory of mind: A meta-analysis of functional brain imaging studies. *Neuroscience & Biobehavioral Reviews* **42**, 9-34, doi:<https://doi.org/10.1016/j.neubiorev.2014.01.009> (2014).
- 17	Wilson, A. C. Do animated triangles reveal a marked difficulty among autistic people with reading minds? *Autism* **25**, 1175-1186, doi:10.1177/1362361321989152 (2021).
- 18	Dennett, D. C. *The Intentional Stance*. (The MIT Press, 1989).
- 19	Abell, F., Happe, F. & Frith, U. Do triangles play tricks? Attribution of mental states to animated shapes in normal and abnormal development. *Cognitive Development* **15**, 1-16, doi:10.1016/S0885-2014(00)00014-9 (2000).
- 20	White, S. J., Coniston, D., Rogers, R. & Frith, U. Developing the Frith-Happé animations: A quick and objective test of Theory of Mind for adults with autism. *Autism Research* **4**, 149-154, doi:<https://doi.org/10.1002/aur.174> (2011).
- 21	Castelli, F., Happé, F., Frith, U. & Frith, C. Movement and Mind: A Functional Imaging Study of Perception and Interpretation of Complex Intentional Movement Patterns. *Neuroimage* **12**, 314-325, doi:<https://doi.org/10.1006/nimg.2000.0612> (2000).
- 22	Castelli, F., Frith, C., Happé, F. & Frith, U. Autism, Asperger syndrome and brain mechanisms for the attribution of mental states to animated shapes. *Brain* **125**, 1839-1849, doi:10.1093/brain/awf189 (2002).
- 23	Livingston, L. A., Shah, P. & Happé, F. *Further developing the Frith-Happé animations: A quicker, more objective, and web-based test of theory of mind for autistic and neurotypical adults* (2019).
- 24	Diamond, A. Executive functions. *Annu Rev Psychol* **64**, 135-168, doi:10.1146/annurev-psych-113011-143750 (2013).
- 25	McElreath, R. *Statistical Rethinking : A Bayesian Course with Examples in R and STAN*. (CRC Press LLC, 2020).
- 26	Khan, A. *et al.* Environmental pollution is associated with increased risk of psychiatric disorders in the US and Denmark. *PLOS Biology* **17**, e3000353, doi:10.1371/journal.pbio.3000353 (2019).
- 27	Hadjiosif, A. M., Morehead, J. R. & Smith, M. A. A double dissociation between savings and long-term memory in motor learning. *PLOS Biology* **21**, e3001799, doi:10.1371/journal.pbio.3001799 (2023).
- 28	Suzuki, S. *et al.* Individuals with problem gambling and obsessive-compulsive disorder learn through distinct reinforcement mechanisms. *PLOS Biology* **21**, e3002031, doi:10.1371/journal.pbio.3002031 (2023).
- 29	Abu-Akel, A. & Shamay-Tsoory, S. Neuroanatomical and neurochemical bases of theory of mind. *Neuropsychologia* **49**, 2971-2984, doi:<https://doi.org/10.1016/j.neuropsychologia.2011.07.012> (2011).
- 30	Orso, B. *et al.* Anatomical and neurochemical bases of theory of mind in de novo Parkinson's Disease. *Cortex* **130**, 401-412, doi:<https://doi.org/10.1016/j.cortex.2020.06.012> (2020).
- 31	Lawrence, A. D., Calder, A. J., McGowan, S. W. & Grasby, P. M. Selective disruption of the recognition of facial expressions of anger. *NeuroReport* **13** (2002).
- 32	Barnby, J. M., Bell, V., Deeley, Q. & Mehta, M. A. Dopamine manipulations modulate paranoid social inferences in healthy people. *Translational Psychiatry* **10**, 214, doi:10.1038/s41398-020-00912-4 (2020).
- 33	Mikus, N. *et al.* Blocking D2/D3 dopamine receptors in male participants increases volatility of beliefs when learning to trust others. *Nature Communications* **14**, 4049, doi:10.1038/s41467-023-39823-5 (2023).
- 34	Sui, J., He, X. & Humphreys, G. W. Perceptual effects of social salience: Evidence from self-prioritization effects on perceptual matching. *Journal of Experimental Psychology: Human Perception and Performance* **38**, 1105-1117, doi:10.1037/a0029792 (2012).
- 35	Sui, J. & Humphreys, G. W. The Integrative Self: How Self-Reference Integrates Perception and Memory. *Trends in Cognitive Sciences* **19**, 719-728, doi:10.1016/j.tics.2015.08.015 (2015).
- 36	Forbes, P. A. G., Korb, S., Radloff, A. & Lamm, C. The effects of self-relevance vs. reward value on facial mimicry. *Acta Psychologica* **212**, 103193, doi:<https://doi.org/10.1016/j.actpsy.2020.103193> (2021).
- 37	Adams, R., Stephan, K., Brown, H., Frith, C. & Friston, K. The Computational Anatomy of Psychosis. *Frontiers in Psychiatry* **4**, doi:10.3389/fpsyt.2013.00047 (2013).
- 38	Mikus, N. *et al.* Effects of dopamine D2 and opioid receptor antagonism on the trade-off between model-based and model-free behavior in healthy volunteers. *bioRxiv*, 2022.2003.2003.482871, doi:10.1101/2022.03.03.482871 (2022).
- 39	Conway, J. R., Catmur, C. & Bird, G. Understanding individual differences in theory of mind via representation of minds, not mental states. *Psychonomic Bulletin & Review* **26**, 798-812, doi:10.3758/s13423-018-1559-x (2019).
- 40	Cools, R. & D'Esposito, M. Inverted-U–Shaped Dopamine Actions on Human Working Memory and Cognitive Control. *Biological Psychiatry* **69**, e113-e125, doi:<https://doi.org/10.1016/j.biopsych.2011.03.028> (2011).
