# Supplementary material for: Disruption of dopamine D2/D3 system function impairs the human ability to understand the mental states of other people
Source: PLoS Biol. 2024 Jun 13;22(6):e3002652. doi: 10.1371/journal.pbio.3002652 (PMC11175582; doi:10.1371/journal.pbio.3002652)
Supplement: Attachment — Submitted filename: R2R_2.docx [file pbio.3002652.s012.docx]

REVIEWS:

Reviewer #2: Reviewer comments

The author responses to my earlier comments were excellent, and unless stated otherwise, I find their responses satisfactory. As mentioned before, the study's strengths lie in its within-subject design and robust human pharmacological methods. However, I still have some lingering concerns about the conclusions drawn in this paper. In this follow-up evaluation, I'll address the claims made by the authors in the order presented and the evidence supporting those claims. Some of this may overlap with my previous review if certain points weren't adequately addressed.

Summary:

Each conclusion presents its own set of challenges. The primary conclusion establishes that dopamine manipulation can impact mental state attribution across various animated scenarios, but it lacks significant insight into the underlying mechanism or specificity of effect for social cognition that would take us a significant step forward. On the other hand, the secondary conclusion relies on a questionable distinction between video categories without robust theoretical support for the tests and with questionable statistical inferences. I genuinely wish I could be more positive, but it's challenging to fully endorse the conclusions as they are.

More detail:

We still need a Bayesian's oversight on the confidence intervals question (but also the lack of correction for multiple comparisons).

We hope Reviewer 4’s evaluation of our use of Bayesian methods will convince Reviewer 2 and/or the editors that our results are valid.

As for the second part of this comment, we would like to point the reviewers/editors to this article by Andrew Gelman & colleagues ‘Why we (usually) don’t have to worry about multiple comparisons’^1^:

In this paper Gelman argues that when using Bayesian statistics, in particular Bayesian multilevel models, we usually do not require multiple comparison correction. (1) In contrast to null hypothesis significance testing (NHST), which assumes the H0 as alternative to the research hypotheses, from the Bayesian perspective researchers very rarely believe that the H0 is strictly true. Gelman et al. have therefore suggested to frame the multiple comparisons issue in terms of sign (S-) and magnitude (M-) errors. (2) Multilevel models serve to minimise these errors through shrinkage, because individual estimates are not only informed by individual data but also by overall group estimates, with partial pooling of the data towards the group mean. Shrinkage helps drastically reduce uncertainty around estimates, which is the main reason why researchers are worried about multiple comparisons in the first place.

The primary finding here is the observed decrease in the accuracy of animations labeling with the administration of haloperidol, which suggests a connection to dopamine in Theory of Mind (ToM). After replicating this effect in a mixed model with the shared dataset, I do agree that the basic effect is indeed present. It's great that the authors made some qualifications in the discussion, which I appreciate.

However, the primary claim of a causal role in ToM still takes the spotlight in the abstract. It might be helpful to acknowledge that this assertion could potentially mislead readers, as we discussed in my previous review. Since there isn't enough evidence to directly link dopamine modulation to ToM, it's important to be cautious in interpreting the findings and adjust the language accordingly throughout the manuscript. For example, using phrasing in the abstract similar to the later statement on lines 437-439 "Our findings thus demonstrate that dopaminergic pathways impact [changed from involved in] Theory of Mind, at least indirectly," would more accurately reflect the scope of the evidence presented.

We thank the reviewer for this suggestion and have changed the wording in the abstract to reflect the scope of the evidence presented in our manuscript more appropriately:

“We conclude that dopaminergic pathways impact Theory of Mind, at least indirectly.”

I also noticed that some labels in the provided dataset aren't very clear. It might be beneficial to include a glossary to explain terms like 'Halday' and 'drugday' to avoid any confusion, especially since their distinctions remain ambiguous despite differences in values, and accuracy differences aren't apparent in the datasets.

At the reviewer’s recommendation we have uploaded a glossary ‘Dopamine challenge reduces mental state attribution accuracy - metadata.pdf’ to <https://osf.io/xm7ty/> where we explain all relevant data and terms. To avoid confusion we have additionally removed all variables from the dataset that were not used in the analyses, such as ‘HALday’.

With these adjustments made throughout the manuscript, the conclusion becomes acceptable. However, standing alone, I'm not entirely convinced it meets the criteria of exceptional importance and interest to scientists set by PLOS. We can probably assume even without this experiment that dopamine manipulations affecting cognition (like attention, motivation, and learning) will impact ToM tasks. So, the paper's significance might hinge on uncovering the mechanisms behind dopamine's impact, which the authors attempt to demonstrate with limited success in the secondary analyses. Other reviewers may disagree. Some might believe that it is important to confirm that there is an effect, even though it is likely there would be.

The secondary conclusion is that 'dopamine modulates inference from mental- and non-mental state animations via independent mechanisms' based on two lines of work. It seems like the authors are diving into some exploratory territory, which can be exciting but also raises some conventional but considerable questions. For instance, why would our motor responses or emotion recognition be more relevant to understanding intentions in the 'mental state' category compared to the 'non-mental state' one?  It's not immediately clear from a theoretical standpoint. Moreover, the Bayesian approach also makes it difficult to control for multiple comparisons.

We thank the reviewer for this comment. We argue that it is possible that the non-mental state animations were sufficiently easy for participants to interpret, and therefore may not have required simulation processes to make sense of the scenario. In other words, other informative motion cues such as the time-courses of both triangles’ movements (e.g., following: both triangles move at the same time and in same direction; fighting: both triangles move simultaneously but in opposite directions, ‘facing’ each other) or the relative distance between triangles may have effectively conveyed the respective narrative, not requiring participants to recruit their own kinematic representations of the triangles’ movements.

The first claim is that haloperidol diminishes the specificity of motor similarity use for the 'mental state' category attribution accuracy.  This diminishing effect is considered by the authors to be a specific reduction of the use of 'motor similarity' for the 'mental state' condition (a contrast between PLA and HAL). Was that comparison done? I could not find it. 

For consideration, I tested the reported 3-way interaction with a (frequentist) linear mixed model equivalent of the analysis, and it is not significant (p = 0.076, regardless of method). Frustratingly, there is no way to provide a regression table in a review. It becomes less significant if the mental state is added as a random slope or if the video is not a random grouping factor (this is not the best fitting model presumably, but it just suggests the finding may not be the most reliable). So, I consider this effect with some skepticism, but there is probably a trend effect here. I do not wish to open a Bayesian vs frequentist debate; I just think the authors and editors consider that the two statistical approaches disagree, and what should a reader do with that information when the study was not preregistered.

We assume the reviewer is referring to model 2.1. In model 2.1, a 3-way interaction between the effect of jerk difference, mental state condition and drug condition was found. While the positive coefficient for this contrast (jerk difference, mental state condition and drug condition), as well as the post-hoc models, already indicate a difference in the jerk difference effect between placebo and haloperidol conditions, we appreciate that further contrasts are justified here and have added the specific contrasts for PLA-HAL differences to the manuscript. While credible intervals indicate some uncertainty around the contrast estimate, the results do point towards a large difference in the slope of jerk difference between placebo and haloperidol conditions within mental state animations, whereas no such drug-placebo difference emerges for non-mental animations. We have updated the manuscript accordingly:

Lines 300-304: “[…] *under haloperidol, this negative effect was diminished (*${E\mu}_{jerkDiff,mentalVSnon-mental,HALvsPLA}$ *= 0.68, CrI = [-0.12, 1.47],* $P({E\mu}_{jerkDiff,HALvsPLA,mentalVSnon-mental}>0)$ *= 0.95; Contrasts of jerk difference slope HAL vs. PLA - non-mental state:* $E\mu$ *= 0.06, CrI = [-0.26, 0.38], mental state:* $E\mu$ *= 0.74, CrI = [-0.03, 1.44]).*”

Now, onto the graphs. I noticed something interesting in Figure 2—the jerk difference in the 'mental state' condition seems to hit a ceiling around z = 5.3, while the 'non-mental state' condition doesn't show the same pattern. What's going on there? Does this difference in distribution affect the analysis?

The range in jerk difference is higher for non-mental state animations because these animations have higher variation in jerk (with following animations mostly depicted with low jerk, and fighting ones depicted with high jerk). To our knowledge, the only way that unequal variances in jerk difference across the two levels of mental state would affect the analysis is if they result in unequal residual variances, thereby violating the assumption of homoskedasticity of linear models. As can be seen in the published R script, we have visually checked for unequal error variances of all our models and could not identify any issues with model 2.1. Please see below for a figure which can be produced from the R script, showing the relationship between predicted (x-axis) and residual (y-axis) values, with no signs of heteroskedasticity.

On lines 341-347 (Model 3, placebo jerk), the authors suggest that due to the absence of an interaction [between jerk difference (motor similarity), mental state, and drug], an effect of placebo jerk difference on mental state animations can be inferred for both placebo (PLA) and haloperidol (HAL) trials. Specifically, they suggest that under haloperidol administration, participants' attribution accuracy is influenced by their movements in the placebo condition rather than movements produced under the drug condition. The authors do need to be careful here. The absence of a difference between HAL and PLA conditions does not signify their equivalence. As proponents of Bayesian analysis, the authors could ascertain whether sufficient evidence exists for the absence of a difference between PLA and HAL conditions to make that inference. Additionally, an investigation solely within the HAL condition to determine if placebo-jerk difference serves as a significantly superior predictor compared to HAL-jerk difference is warranted. As it stands, Figure 2C looks very similar to Figure 2B. By completing those analyses the authors could solidify their conclusions: that HAL-treated participants rely more on placebo-based motor inferences than HAL-based ones, and similar to placebo.

We thank the reviewer for this comment and hope we can provide more confidence that indeed we have found evidence for absence of an effect of haloperidol jerk difference (movements affected by the drug) within the haloperidol condition with the following results:

First, the ‘hypothesis’ function of the brm package returns the evidence ratio (i.e., ratio of posterior to prior probability, equivalent to the Bayes factor) of an effect. Here, values greater than one indicate that the evidence in favor of the hypothesis is larger than the evidence against that hypothesis. The evidence ratio for the hypothesis that the 3-way interaction of drug, mental state and placebo jerk difference is zero is 4.46.

Second, as requested by the reviewer, we ran a further model (Model 3.2) with only HAL trials and both placebo and haloperidol jerk difference as parameters in the model. While the model clearly shows no effect for haloperidol jerk difference in mental state animations (${E\mu}_{HALjerkDiff,mentalVSnon-mental}$ = -0.30, CrI = [-1.14, 0.50]), the effect of placebo jerk difference in this model is even stronger and more robust than in the previous model (Model 3): ${E\mu}_{PLAjerkDiff,mentalVSnon-mental}$ = -0.70, CrI = [-1.38, -0.03].

We have added the following lines to support our claim in the manuscript:

Lines 365 – 373: “To further corroborate this finding that placebo-, and not haloperidol jerk difference affected accuracy in both PLA and HAL trials, we ran a second model (Model 3.2) with only HAL trials and both placebo and haloperidol jerk difference as predictors. This model clearly showed no effect of haloperidol jerk difference (${E\mu}_{HALjerkDiff,non-mental}$ = 0.03, CrI = [-0.35, 0.40]; ${E\mu}_{HALjerkDiff,mentalVSnon-mental}$ = -0.30, CrI = [-1.14, 0.50]), and an even stronger effect of placebo jerk difference (${E\mu}_{PLAjerkDiff,non-mental}$ = -0.06, CrI = [-0.44, 0.31]; ${E\mu}_{PLAjerkDiff,mentalVSnon-mental}$ = -0.70, CrI = [-1.38, -0.03], $P\left( {E\mu}_{PLAjerkDiff,mentalVSnon-mental}<0 \right)$ = 0.98, coefficient for placebo jerk difference in mental state animations = -0.06 + (- 0.70) = -0.76) on mental state animations*.*”

The additional analyses can be found in the updated version of the .rmd script on <https://osf.io/xm7ty/>.

Including HAL jerk differences in the online data could really help when it comes to reviewing the information more closely.

We would like to make the reviewer aware that HAL jerk differences (as well as the PLA jerk differences) are already included in the data provided online, as they are needed to run models 2.1 and 2.3.

Another point of the authors supporting the claim about two different paths is the connection to emotion regulation. On line 383, the authors mention, "If we see specific changes in how drugs affect recognizing emotions and understanding mental states, but not for non-mental state animations, it could show how dopamine affects our social understanding." However, I respectfully disagree with this because both types of videos likely involve our social understanding, as I noted in my earlier review.

When we look at the secondary analyses together, it seems that haloperidol mainly affects the 'mental state' category and related measures. This includes both individual differences in how we perceive ourselves and others, as well as recognizing emotions. Initially, I thought the abstract suggested they found separate effects for each category, but it turns out they both hinge on distinguishing between 'mental state' and 'non-mental state'. Could it be that haloperidol affects motivation or effort rather than independent mechanisms? Especially since the 'mental state' category is known to be more challenging and might require more focus?

My more fundamental issue with the secondary conclusions is that both categories require understanding mental states, and the line between them isn't clear. The authors did a great job summarizing previous research on these conditions. While I can see that they might involve subtly different levels of mentalizing, labeling them as 'mental state' and 'non-mental state' will cause confusion. Typically, the literature compares tasks involving mentalizing to those that don't, rather than two tasks with slight differences in mentalizing. Also, given that both categories involve inferring mental states, it might be misleading to suggest that 'non-mental state' tasks don't involve understanding others' minds. This is what is implied by the authors on line 383 (that only one category involves social cognition).

My main concern is that there doesn't seem to be a clear theoretical difference between the categories. Without well-defined criteria, it's hard to claim separate pathways for different categories, as the authors suggest. Instead, the differences in effects might reflect varying levels of effort, attention, or other factors. Upon reflection, it's worth considering that these animation categories differ in various ways, like how the triangles interact, move, and demand attention. These differences might explain why one task is easier than another and its relation to other tasks.

One solution could be to reconsider the labels used or even drop this comparison altogether. It might be best to do so if it's unclear why these categories differ in terms of participants' cognition.  That said, I appreciate that the primary finding may not be compelling enough on its own, so the authors may want to try to flesh out what is really different between these video categories, and go from there -- as long as they can justify the results in terms of exploratory analysis (without correction for multiple comparisons, if appropriate etc).

We thank the reviewer for their comments here and understand the reservations. In response we now outline more clearly (1), our justification for a clear theoretical difference between the mental- and non-mental state animations and (2), empirical evidence supporting this theoretical difference.

(1): The theory underlying our hypotheses and conclusions is rooted in philosophy of mind (in particular, Daniel Dennett’s work^2^). The particular proposal made by the theory is that the key distinction between the mental- and non-mental state categories is that the mental-state animations cannot be decoded without recognising that one agent (triangle) is causing, or acting upon, mental states in the other. In contrast, the non-mental state animations can be decoded accurately without recognising these propositional properties.

(2): In addition to the empirical evidence we have already mentioned in our last rebuttal, there are further findings from clinical work clearly distinguishing the non-mental from the mental state animations: While verbal responses by control participants describing the animations show clear distinctions between the non-mental- and mental state animations (with mental state animations being described with rich, mentalistic language in contrast to non-mental state animations, which are generally described using mainly movement terms), autistic participants’ responses do not show these distinctions, and their descriptions of mental state animations lack the mentalistic language seen in control participants. We argue that these differences between clinical and control groups clearly reflect a difference in the tendency / ability to identify and attribute mental states to the triangles and provide empirical support for the theoretical distinction.

Lastly, regarding the effectiveness of blinding, was there any way to assess this?

We have debrief sheets for the study but have not analysed them yet as it is unclear exactly how we would do this and how much it would shape the conclusions. However, if it is deemed worthwhile, we are happy to look at this data.

On line 41, it might be worth noting that there's no 'published' study on this topic.

We have edited our manuscript to acknowledge that there may be studies on this topic which have not been published.

Lines 39 – 42: “*While a handful of existing studies show effects of dopamine D2 receptor antagonism on emotion recognition^3,4^ and social belief updates^5-7^, to the best of our knowledge, no published study to date has explored effects of dopamine manipulation on ToM function in healthy individuals*.”

Reviewer #3: Thankyou to the authors for responding to all of my comments. I am happy that they have all been addressed.

Reviewer #4:
I have been asked to review this revision with a special focus on the Bayesian analyses. In brief, I thought the analyses were entirely appropriate. You might consider orientating the reader to the nature of your inference with something like the following:

"In what follows, we report our findings in terms of Bayesian credible intervals and the posterior probability of models with and without an effect of interest (e.g., main effects or interactions). In brief, we used a standard analysis package to assess the evidence (a.k.a., marginal likelihood) for alternative models using a leave one out cross validation scheme. Crucially, using plausible (mildly informative) priors over random effects, this kind of analysis eludes a point null hypothesis — and allows us to specify, with a certain confidence, whether an effect was present or absent."

We thank the reviewer for this excellent suggestion and have incorporated it in the manuscript, to better reflect the fact that using Bayesian methods, our inferences are not driven by binary H1 vs. H0 testing, but rather are represent the probability of an effect being present:

Lines 196 – 208: “*In what follows, we report our findings in terms of Bayesian credible intervals and the posterior probability of models with and without an effect of interest (e.g., main effects or interactions). In brief, we used a standard analysis package (brms^8^) to assess the evidence for alternative models using a leave one out cross validation scheme (using the LOO^9^ function). Crucially, using plausible (mildly informative) priors over random effects, this kind of analysis eludes a point null hypothesis — and allows us to specify, with a certain confidence, whether an effect was present or absent. This confidence is reflected by the 95% credible intervals (CrIs^[1]^), as well as the posterior probability that a certain effect (*$E\mu$*) is different from zero (*$P(E\mu<0)$*) /* $P(E\mu>0)$*). Consequently, for all relevant model parameters, we report expected values under the posterior distribution and their CrIs, as well as their posterior probabilities. In line with Franke & Roettger^11^, we conclude there is compelling evidence for an effect if its posterior probability* $P\left( E\mu\neq0 \right)$ *is close to 1*.”

Minor points

Line 35, you might replace "An incisive way" with "An unambiguous way"

We thank the reviewer for this suggestion but have remained with ‘incisive’ in this case, as we feel it more closely reflects what we intend to say.

Line 109: could you replace "after drug administration" with "after drug or placebo administration". Most readers will associate the drug with Haloperidol.

As per the reviewer’s suggestion, we have added ‘or placebo’ to the sentence starting in Line 109.

Line 156: when you say that " participants viewed and rated" the animations, you should explain to the reader what you mean by "rated". Where each of the four words rated individually? Was a rating scale used? Perhaps you could say:

"For example, after each animation, subjects were asked to…"

We have added additional information on how the ratings were conducted:

Lines 158 – 160: “*Following each animation, participants rated on four separate visual-analogue scales (ranging from 1 [not at all] to 10 [very]) how much they thought the last viewed stimulus depicted the target, as well as each non-target word.”*

Line 200: I would replace the "/" between the probabilities with "or". Otherwise, people might think that this is an odds ratio.

We thank the reviewer for spotting this and have amended the manuscript accordingly:

Lines 203 – 205: “*This confidence is reflected by the 95% credible intervals (CrIs^[2]^), as well as the posterior probability that a certain effect (*$E\mu$*) is different from zero (*$P(E\mu<0)$*) or* $P(E\mu>0)$*).*”

I hope these comments help should any revision be required.

References:

1	Gelman, A., Hill, J. & Yajima, M. Why We (Usually) Don't Have to Worry About Multiple Comparisons. *Journal of Research on Educational Effectiveness* **5**, 189-211 (2012). <https://doi.org:10.1080/19345747.2011.618213>

2	Dennett, D. C. in *Machiavellian intelligence: Social expertise and the evolution of intellect in monkeys, apes, and humans.* 180-202 (Clarendon Press/Oxford University Press, 1988).

3	Schuster, B. A. *et al.* Dopaminergic Modulation of Dynamic Emotion Perception. *The Journal of Neuroscience* **42**, 4394 (2022). <https://doi.org:10.1523/JNEUROSCI.2364-21.2022>

4	Lawrence, A. D., Calder, A. J., McGowan, S. W. & Grasby, P. M. Selective disruption of the recognition of facial expressions of anger. *NeuroReport* **13** (2002).

5	Barnby, J. M., Bell, V., Deeley, Q. & Mehta, M. A. Dopamine manipulations modulate paranoid social inferences in healthy people. *Translational Psychiatry* **10**, 214 (2020). <https://doi.org:10.1038/s41398-020-00912-4>

6	Mikus, N. *et al.* Blocking D2/D3 dopamine receptors in male participants increases volatility of beliefs when learning to trust others. *Nature Communications* **14**, 4049 (2023). <https://doi.org:10.1038/s41467-023-39823-5>

7	Rybicki, A. J., Sowden, S. L., Schuster, B. A. & Cook, J. L. Dopaminergic challenge dissociates learning from primary versus secondary sources of information. *bioRxiv*, 2021.2012.2001.470554 (2021). <https://doi.org:10.1101/2021.12.01.470554>

8	Bürkner, P.-C. brms: An R Package for Bayesian Multilevel Models Using Stan. *2017* **80**, 28 (2017). <https://doi.org:10.18637/jss.v080.i01>

9	Vehtari, A., Gelman, A. & Gabry, J. Practical Bayesian model evaluation using leave-one-out cross-validation and WAIC. *Statistics and Computing* **27**, 1413-1432 (2017). <https://doi.org:10.1007/s11222-016-9696-4>

10	Nalborczyk, L., Batailler, C., Lœvenbruck, H., Vilain, A. & Bürkner, P. C. An Introduction to Bayesian Multilevel Models Using brms: A Case Study of Gender Effects on Vowel Variability in Standard Indonesian. *J Speech Lang Hear Res* **62**, 1225-1242 (2019). <https://doi.org:10.1044/2018_jslhr-s-18-0006>

11	Franke, M. & Roettger, T. *Bayesian regression modeling (for factorial designs): A tutorial* (2019).

1. The term credible interval comprises the Bayesian analogue of a classical confidence interval, except that probability statements can be made based upon it (see 10	Nalborczyk, L., Batailler, C., Lœvenbruck, H., Vilain, A. & Bürkner, P. C. An Introduction to Bayesian Multilevel Models Using brms: A Case Study of Gender Effects on Vowel Variability in Standard Indonesian. *J Speech Lang Hear Res* **62**, 1225-1242 (2019). https://doi.org:10.1044/2018_jslhr-s-18-0006)
2. The term credible interval comprises the Bayesian analogue of a classical confidence interval, except that probability statements can be made based upon it (see 10	ibid.)
